# Theoretical studies on the photophysical properties of luminescent pincer gold(iii) arylacetylide complexes: the role of π-conjugation at the C-deprotonated [C^N^C] ligand†

**DOI:** 10.1039/c4sc03697b

**Published:** 2015-03-10

**Authors:** Glenna So Ming Tong, Kaai Tung Chan, Xiaoyong Chang, Chi-Ming Che

**Affiliations:** a State Key Laboratory of Synthetic Chemistry, Institute of Molecular Functional Materials, Department of Chemistry, The University of Hong Kong Pokfulam Road Hong Kong SAR China tongsm@hku.hk cmche@hku.hk; b HKU Shenzhen Institute of Research and Innovation Shenzhen 518053 China cmche@hku.hk

## Abstract

We have performed theoretical analyses of the photophysical properties of a series of cyclometalated gold(iii) arylacetylide complexes, [(C^N^C)Au^III^C≡CPh-4-OMe], with different extents of π-conjugation at the doubly C-deprotonated [C^N^C] ligand *via* replacement of one of the phenyl moieties in the non-conjugated C_H_^N^C ligand (1) by a naphthalenyl (2) or a fluorenyl moiety (3-exo and 3-endo; HC_H_^N^CH = 2,6-diphenylpyridine). Conforming to the conventional wisdom that extended π-conjugation imposes rigidity on the structure of the ^3^IL(ππ*(C^N^C)) excited state (IL = intraligand), the calculated Huang–Rhys factors for the ^3^IL → S_0_ transition follow the order: 1 > 2 > 3-exo ∼ 3-endo, which corroborates *qualitatively* the experimental non-radiative decay rate constants, *k*_nr_: 1 ≫ 2 > 3-exo, but not 3-endo. Density Functional Theory (DFT) calculations revealed that there is an additional triplet excited state minimum of ^3^LLCT character (LLCT = ligand-to-ligand charge transfer; ^3^[π(C≡CPh-4-OMe) → π*(C^N^C)]) for complexes 1 and 3-endo. This ^3^LLCT excited state, possessing a large out-of-plane torsional motion between the planes of the C^N^C and arylacetylide ligands, has a double minimum anharmonic potential energy surface along this torsional coordinate which leads to enhanced Franck–Condon overlap between the ^3^LLCT excited state and the ground state. Together with the larger spin–orbit coupling (SOC) and solvent reorganization energy for the ^3^LLCT → S_0_ transition compared with those for the ^3^IL → S_0_ transition, the calculated *k*_nr_ values for the ^3^LLCT → S_0_ transition are more than 690- and 1500-fold greater than the corresponding ^3^IL → S_0_ transition for complexes 1 and 3-endo respectively. Importantly, when this ^3^LLCT → S_0_ decay channel is taken into consideration, the non-radiative decay rate constant *k*_nr_ could be reproduced *quantitatively* and in the order of: 1 ≫ 3-endo, 2 > 3-exo. This challenges the common view that the facile non-radiative decay rate of transition metal complexes is due to the presence of a low-lying metal-centred ^3^dd or ^3^LMCT excited state (LMCT = ligand-to-metal charge transfer). By analysis of the relative order of MOs of the chromophoric [C^N^C] cyclometalated and arylacetylide ligands, one may discern why complexes 1 and 3-endo have a low-lying ^3^LLCT excited state while 3-exo does not.

## Abstract 2

The facile non-radiative decay for gold(iii) complexes is due to the thermally accessible ^3^LLCT, but not the usually assumed ^3^dd excited state.

## Graphical abstract

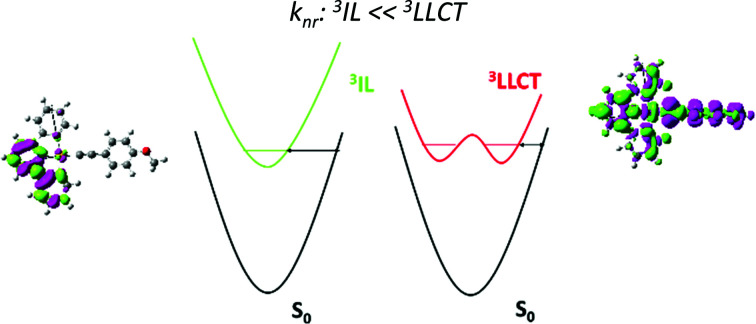

## Introduction

Gold(iii) complexes are being actively studied as potential anticancer drugs^1^ and catalysts.^2,3^ However, the study of the spectroscopic and luminescent properties of gold(iii) complexes is still in its infancy, in particular when compared to their isoelectronic platinum(ii) counterparts, which are known to display rich photophysical behaviours. One of the impediments to the progress of photoluminescence of gold(iii) complexes is the high electrophilicity of the gold(iii) ion and the presence of a low-lying Au(5dσ*) orbital. In effect, the deactivating ligand-to-metal charge transfer (LMCT) and/or dd ligand-field excited states become close in energy to the emitting excited state, leading to efficient luminescence quenching in gold(iii) complexes.^4^ To circumvent this problem, Yam and co-workers have coupled various strong σ-donating ligands, such as arylacetylide and *N*-heterocyclic carbenes (NHC), to the gold(iii) cyclometalated complexes; these complexes were reported to be weakly emissive in solution (*ϕ* < 0.01) at room temperature.^5^

To enhance the emission quantum yield, the structural distortion between the emitting excited state and the ground state must be minimized, thereby decreasing the non-radiative decay rate.^6^ This can be achieved by designing emitting molecules with highly rigid ligand scaffolds, for example, by extended π-conjugation at the cyclometalated ligand^7^ (see Table 1 for a comparison between the emission quantum yields of selected examples of gold(iii) cyclometalated complexes with different extents of π-conjugation at the [C^N^C] ligand).^3*a*,5,8^ A particularly striking example is the series of gold(iii) complexes with a fluorenyl moiety incorporated into the doubly deprotonated [C^N^C] ligand.^3*a*^ In this case, the room temperature emission quantum yields of the gold(iii) cyclometalated complexes in solution reach 0.58, and the corresponding non-radiative decay rate constant (*k*_nr_) falls to 1.74 × 10^3^ s^−1^ (Table 1, column 5). In other words, *k*_nr_ drops more than four orders of magnitude when one of the phenyl moieties in the non-conjugated C_H_^N^C ligand (Table 1, column 2; HC_H_^N^CH = 2,6-diphenylpyridine) is replaced by a fluorenyl moiety.^3*a*^ Similar enhancement in emission quantum yield has also been reported for fluorene-functionalized cyclometalated platinum(ii) complexes when compared with the non-conjugated C_H_^N^C analogue;^9^ the enhanced luminescence is attributed to the rigid π-conjugated fluorene unit which minimizes structural distortion between the emitting triplet excited state and the ground state.

**Table 1:** Photophysical properties of gold(iii) pincer-type complexes in dichloromethane solution at room temperature. For R = C≡CPh-4-OMe, *n* = 0 and for R = 1,3-dimethylimidazol-2-ylidene, *n* = 1. C_X_^N^C = pincer-type cyclometalated ligand; X = H, np, or fl

	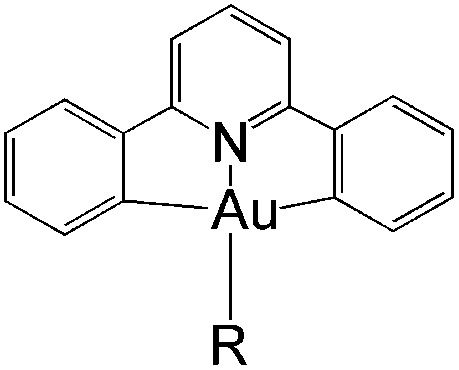	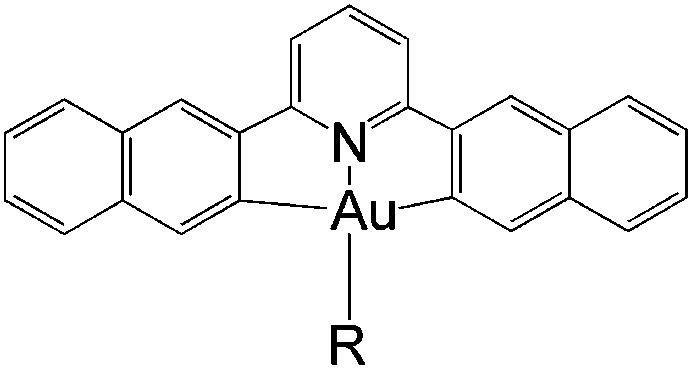	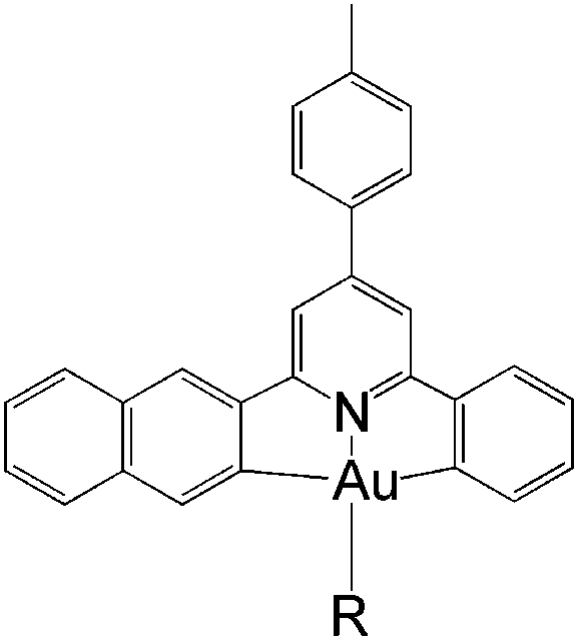	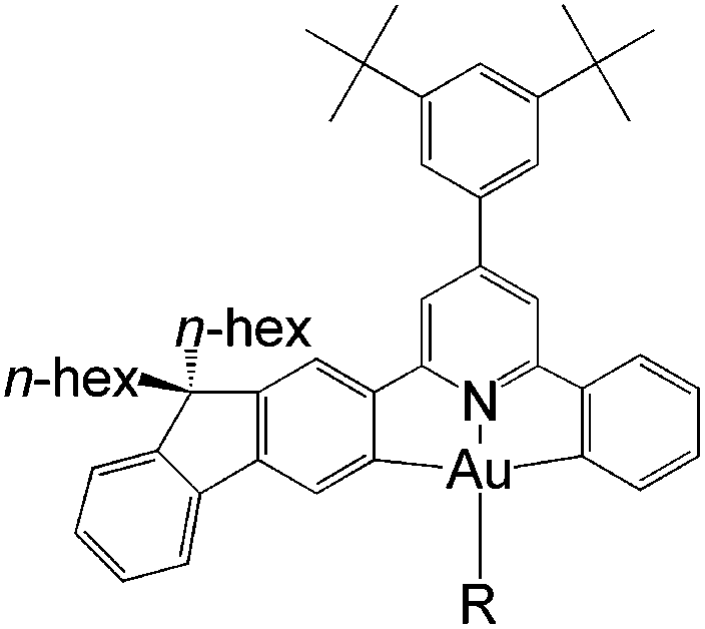	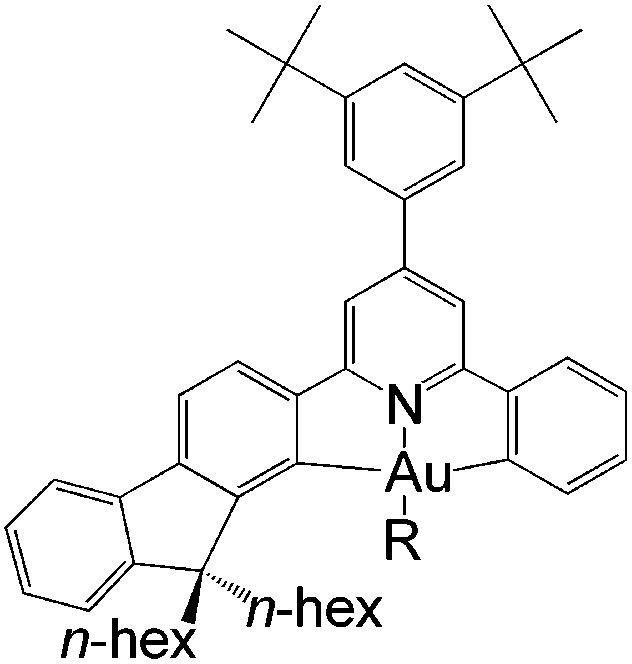
	[Au^III^(C_H_^N^C)R]^*n*^	[Au^III^(C_np_^N^C_np_)R]^*n*^	[Au^III^(C_np_^N^C)R]^*n*^	*exo*-[Au^III^(C_fl_^N^C)R]^*n*^	*endo*-[Au^III^(C_fl_^N^C)R]^*n*^
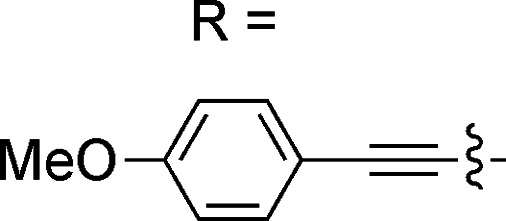	*ϕ* = 0.0004	*ϕ* = 0.08	*ϕ* = 0.09	*ϕ* = 0.58	*ϕ* = 0.02
	*τ* = 0.017 μs^3*a*,5*b*^	*τ* = 64 μs^8*a*^	*τ* = 25 μs^8*a*^	*τ* = 242 μs^3*a*^	*τ* = 14.5 μs (this work)
	*k*_nr_ = 5.88 × 10^7^ s^−1^	*k*_nr_ = 1.44 × 10^4^ s^−1^	*k*_nr_ = 3.64 × 10^4^ s^−1^	*k*_nr_ = 1.74 × 10^3^ s^−1^	*k*_nr_ = 6.76 × 10^4^ s^−1^

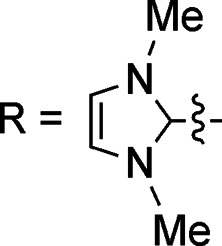	*ϕ* = 0.0039	*ϕ* = 0.055			
	*τ* = 0.6 μs^5*a*^	*τ* = 282 μs^8*b*^			
	*k*_nr_ = 1.66 × 10^7^ s^−1^	*k*_nr_ = 3.35 × 10^3^ s^−1^			

Interestingly, when the fluorenyl moiety is disposed in such a fashion that the long alkyl chains are “*endo*” in the gold(iii) pincer complex (last column in Table 1), there is a dramatic decrease in emission quantum yield (*ϕ* ∼ 0.02, *τ* ∼ 14.5 μs) and a nearly 40-fold increase in the non-radiative decay rate constant (*k*_nr_ ∼ 6.76 × 10^4^ s^−1^) when compared with its “*exo*” analogue (Table 1, column 5; see ESI† for the synthetic procedure and photophysical properties of the “*endo*” complex). This means that, even with a seemingly suitable cyclometalated ligand (*i.e.*, a strong σ-donor which raises the energy of the dd or LMCT excited state and a cyclometalated ligand with extended π-conjugation that minimizes structural distortion), the phosphorescence efficiency of gold(iii) complexes is not necessarily high. Thus, for effective design of functional luminescent molecules, it is important to understand the effect of π-conjugation in the C-deprotonated cyclometalated [C^N^C] ligand on the excited state properties of these luminescent gold(iii) complexes.

**Chart 1:**
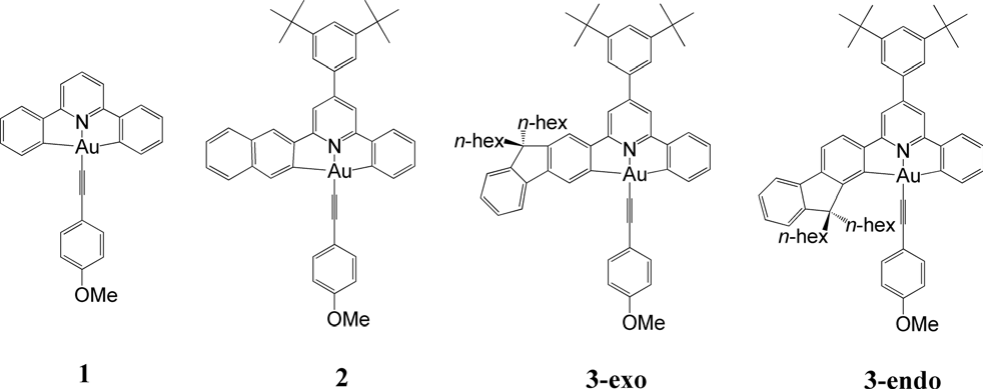


In this work, we have performed a detailed theoretical analysis of four gold(iii) complexes with different [C^N^C] cyclometalated ligand scaffolds (Chart 1), namely, the non-conjugated C_H_^N^C (1) and the π-conjugated C_np_^N^C (2) and C_fl_^N^C (3-exo and 3-endo); complexes 2 and 3-exo (and 3-endo) have one of the phenyl moieties of 1 replaced by a naphthalenyl (np) or a fluorenyl (fl) moiety respectively. The ancillary ligand, *p*-methoxyphenyl acetylide ([C≡CPh-4-OMe]^−^) is kept the same for all four complexes. A detailed list of definitions and abbreviations is provided in the appendix.

## Theoretical background

### Dynamical solvent effect on excited state and ground state energies

Density Functional Theory (DFT) and time-dependent DFT (TDDFT) are the commonly used tools to study the ground state and excited state properties of medium- to large-sized molecules. In the literature, computation of emission energies in solutions is performed using either linear response TDDFT (LR-TDDFT) or the ΔSCF method. For both types of calculations, both the excited state of interest and the ground state are calculated with equilibrium (EQ) solvation. However, in an emission process, the ground state should be treated with solvent polarization in the non-equilibrium (NEQ) regime^10^ because the time scale of an emission process is much faster than that of the solvent dynamics. Therefore, for a rigorous consideration of the solvent effect on an emission process, the ground state should be computed with *non-equilibrium* solvation, *i.e.*, only the solvent electronic polarization (the “fast” component) is in equilibrium with the ground state electron density of the solute, while the solvent nuclear polarization (the “slow” component) remains equilibrated with the excited state electron density of the solute. For this reason, we have employed the state-specific (SS) approach to account for the dynamical solvent effect. Within the SS scheme, rather than using the *ground state* electronic density as in LR-TDDFT and ΔSCF, the electronic density of the *emitting excited state* is used to compute the ground state energy.^10^ Therefore, the emission energy within the SS scheme (Δ*E*^SS^_em_) is given by:

1 Δ*E*^SS^_em_ = *E*^ES^_EQ_(*Q*^ES^_0_) − *E*^GS^_NEQ_(*Q*^ES^_0_)

**Fig. 1:**
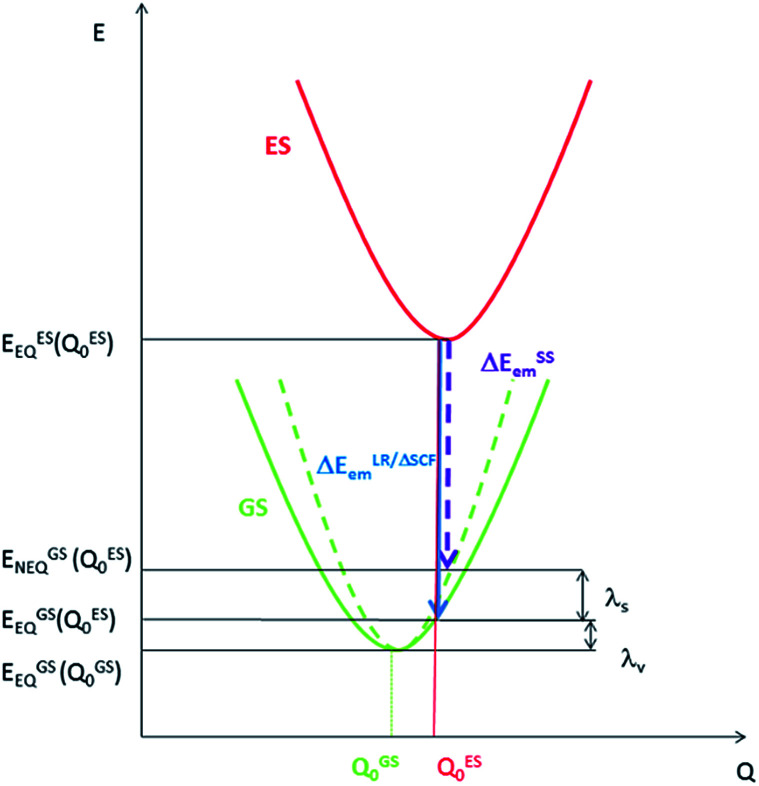
Potential energy surfaces for an electronic transition with energy evaluated with equilibrium solvation (solid line) and non-equilibrium solvation (dashed line).

*E*^ES^_EQ_(*Q*^ES^_0_) is the energy of the excited state (ES) with equilibrium solvation at the optimized excited state geometry (*Q*^ES^_0_), and *E*^GS^_NEQ_(*Q*^ES^_0_) is the energy of the ground state (GS) with non-equilibrium solvation at (*Q*^ES^_0_) (Fig. 1).^11^

The SS approach also allows one to estimate the solvent reorganization energy (*λ*_s_), which is the ground state energy difference calculated with *non-equilibrium* solvation (*E*^GS^_NEQ_(*Q*^ES^_0_)) and with *equilibrium* solvation (*E*^ES^_EQ_(*Q*^ES^_0_)) at the optimized *excited state geometry* (*Q*^ES^_0_) (Fig. 1):^11^

2 *λ*_s_ = *E*^GS^_NEQ_(*Q*^ES^_0_) − *E*^GS^_EQ_(*Q*^ES^_0_)

Similarly, the intramolecular reorganization energy computed within the SS approach (*λ*^SS^_V_) is given by:

3 *λ*^SS^_v_ = *E*^GS^_EQ_(*Q*^ES^_0_) − *E*^GS^_EQ_(*Q*^GS^_0_)

where *E*^GS^_EQ_(*Q*^GS^_0_) is the energy of the ground state computed with *equilibrium* solvation at the optimized *ground state geometry Q*^GS^_0_ (Fig. 1).

### Radiative decay rate constant (*k*_r_)

The total radiative decay rate constant from the vibrational ground state of the emitting T_1_*α*-spin sub-state (*k*_r_^*α*^) to the S_0_ state vibrational manifolds is given by the sum of individual radiative decay rate constants (denoted *k*_r_^*α*^(*ν̃*)), each corresponding to a single vibronic transition, T_1_^*α*^(*υ*′ = 0) → S_0_(*υ*′′), with photon energy, *ν̃*, and vibrational quantum number for the T_1_ and S_0_ states, *υ*′ and *υ*′′, respectively:4
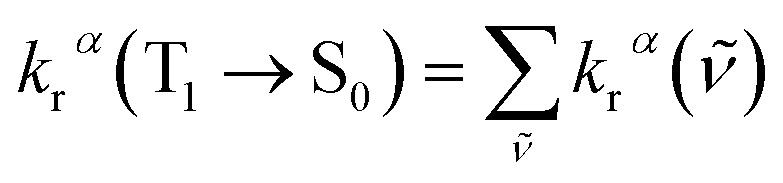


The radiative decay rate constant for the single vibronic transition can be calculated from the Einstein coefficient of spontaneous emission:^12^5
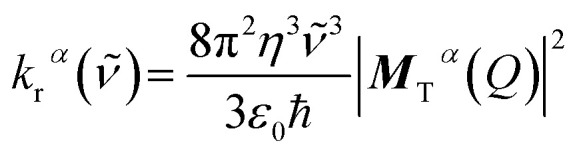
where *η* is the solvent refractive index, *ν̃* is the triplet emission energy (in cm^−1^), and ***M***_T_^*α*^(*Q*) is the transition dipole moment of the T_1_^*α*^ → S_0_ transition (in *ea*_0_), and the prefactor 8π^2^/3*ε*_0_*ħ* = 2.0261 × 10^−6^.

By invoking the Condon approximation (*i.e.*, ***M***_T_^*α*^(*Q*) ≈ ***M***_T_^*α*^(*Q*^T1^_0_) with *Q*^T1^_0_ being the optimized T_1_ excited state geometry) and combining eqn (4) and (5), the total radiative decay rate constant, *k*_r_^*α*^, is given by:^13^6
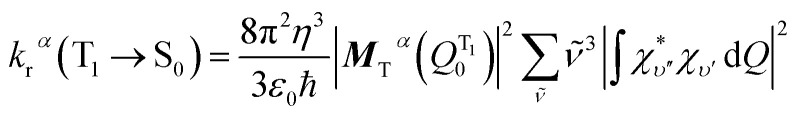
*χ*_*υ*′′_ and *χ*_*υ*′_ are the vibrational wavefunctions of the S_0_ and the T_1_ states respectively.

Unless the emission spectrum is sharply peaked, as in an atomic emission spectrum, one should not take the integral in eqn (6) as unity and replace the summation in eqn (6) by the emission peak maximum, *ν̃*_max_^3^; such an approximation is justified only if the molecule has fixed nuclei. In reality, however, the nuclei are in motion, bringing about a broadening of the emission spectrum. These nuclear motions (*i.e.*, vibrations) can be accounted for by the Franck–Condon factors ((|∫χ^*^_υ′′_*χ*_*υ*′_d*Q*|^2^). In general, one may approximate the last term in the summation as:^13^7
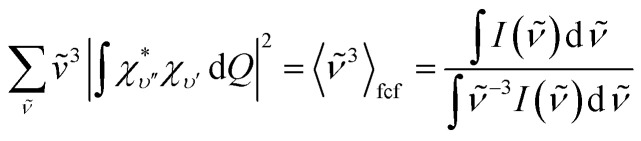
with *I*(*ν̃*) being the emission intensity at *ν̃* (corrected to the number of photons emitted per unit wavenumber). The emission intensity can be obtained either from experiment or by computational simulation. The total radiative decay rate constant for the T_1_^*α*^ → S_0_ transition may then be written as:8
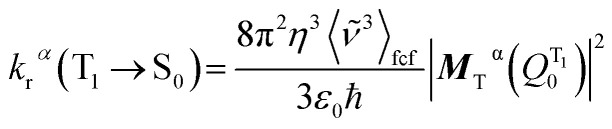


The transition dipole moment ***M***_T_^*α*^(*Q*^T1^_0_) could be obtained by first-order perturbation interactions between the T_1_*α*-spin sub-state and the singlet excited state *via* spin–orbit coupling (SOC):^12^9
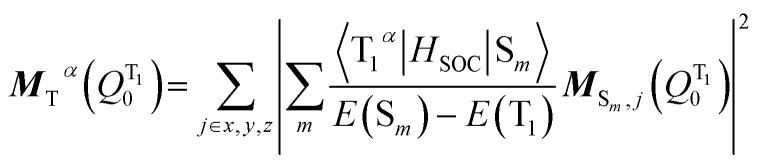
where ***M***_S_*m*_,*j*_ is the *j*-axis projection of the S_*m*_ → S_0_ transition dipole moment, *E*(T_1_) and *E*(S_*m*_) are the energies of the T_1_ and the *m*^th^ singlet (S_*m*_) excited states, respectively, and 〈T_1_^*α*^|*H*_SOC_|S_*m*_〉 are the SOC matrix elements between the T_1_*α*-spin sub-state and the S_*m*_ excited state.

As the energy splitting between the three T_1_*α*-spin sub-states is less than 5 cm^−1^, all sub-states should be equally populated at room temperature. Therefore, the average radiative decay rate constant *k*_r_ is given by:10
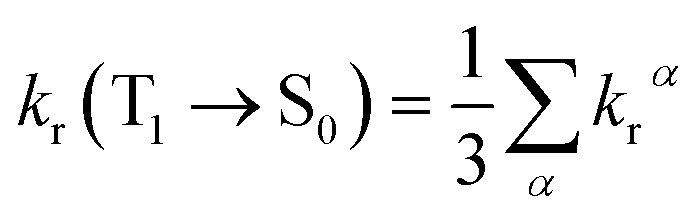


### Non-radiative decay rate constant (*k*_nr_)

In the limit of the Franck–Condon approximation in the non-adiabatic regime, the non-radiative decay rate constant (*k*_nr_) of the T_1_ → S_0_ transition can be estimated by application of the Fermi's Golden Rule expression, assuming that both electronic states are harmonic:^14^11
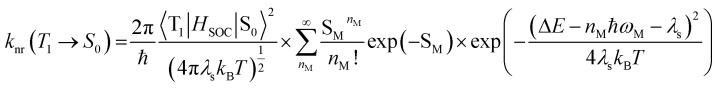


This expression can be applied when *ħω*_M_ ≫ *k*_B_T and the solvent orientational and librational motions are treated classically. *ω*_M_ are the high-frequency (hf) intraligand vibrational modes (*ħω*_M_ > 1000 cm^−1^), typically corresponding to the aromatic CC/CN stretching modes (*ħω*_M_ ∼ 1200–1500 cm^−1^) and C≡C stretching modes (*ħω*_C≡C_ ∼ 2200–2300 cm^−1^) if the acetylide ligand is involved in the complex; *λ*_S_ is the solvent reorganization energy and may be obtained from eqn (2); Δ*E* is given by

12a Δ*E* = Δ*E*_00_ − *λ*_lf_

with Δ*E*_00_ being the zero-point energy difference between the T_1_ and S_0_ states and *λ*_lf_ being the reorganization energy contributed by the low-frequency (lf) modes of the complex (*i.e.*, *ħω*_lf_ < 1000 cm^−1^). Assuming that all the normal modes are harmonic oscillators,12b
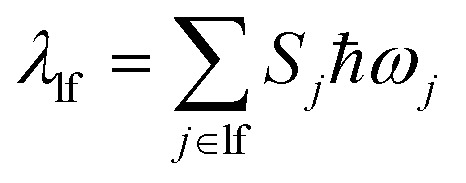
12c
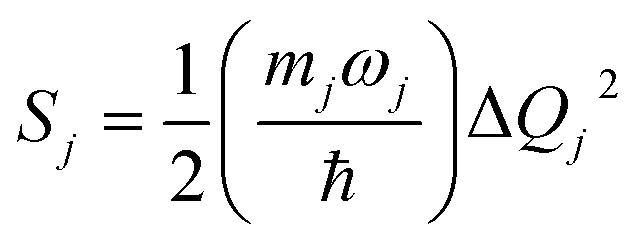
*S*_*j*_, *m*_*j*_, and Δ*Q*_*j*_ are the Huang–Rhys factor, the reduced mass, and the equilibrium displacement of the *j*^th^ normal mode *ω*_j,_ respectively; *S*_M_ and *n*_M_ are the Huang–Rhys factor and the number of quanta of the effective high frequency mode *ħω*_M_ (corrected to the nearest integer), respectively:12d
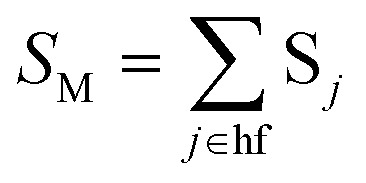
12e
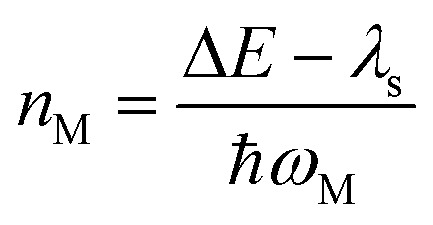


Under the harmonic oscillator approximation, the intramolecular reorganization energy, *λ*^FC^_v_, could be estimated as:13
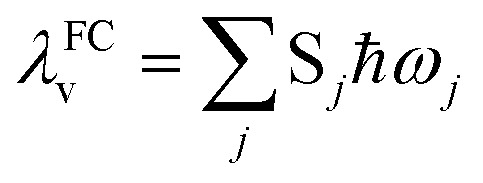
where the summation runs over all the normal modes, *ω*_*j*_.

### Computational details

In this work, the hybrid density functional, PBE0,^15^ was employed for all calculations using the program package G09.^16^ The 6-31G* basis set^17^ was used for all atoms except Au, which was described by the Stuttgart relativistic pseudopotential and its accompanying basis set (ECP60MWB).^18^ The solvent effect was also included by means of the polarizable continuum model (PCM) with the solvent as dichloromethane (CH_2_Cl_2_; *η* = 1.424).^19^ Geometry optimizations of the singlet ground state (S_0_) and the lowest triplet excited state (T_1_) were respectively carried out using restricted and unrestricted density functional theory (*i.e.*, RDFT and UDFT) formalisms without symmetry constraints. Frequency calculations were performed on the optimized structures to ensure that they were minimum energy structures by the absence of imaginary frequency (*i.e.*, NImag = 0). Stability calculations were also performed for all the optimized structures to ensure that all the wavefunctions obtained were stable.

Vertical transition energies were computed using the linear response approximation for absorption, but the state specific approach for emission.^20^ For the radiative decay rate constant calculation (using eqn (8) and (9)), the singlet excited state energy, *E*(S_*m*_), the associated transition dipole moment of the S_*m*_ → S_0_ transition ***M***_S*m,j*_ (*j* = *x*, *y*, *z*), and the coefficients necessary to compute the SOC matrix elements (*i.e.*, the d-orbital coefficients (*c*_d_) of Au in the MO relevant to the coupling excited states and the corresponding CI coefficients), were all obtained from a state-specific approach using “ExternalIteration” implemented in G09.^10*b*,20^

The Huang–Rhys factor *S*_*j*_ (using eqn (12c)) for the normal mode *ω_j_* may be obtained by performing a Franck–Condon calculation implemented in G09 *via* “freq = fc” and “prtmat = 2”. The simulated emission spectrum allows one to calculate the Franck–Condon factor-weighted emission energy 〈*ν̃*〉_fcf_ (using eqn (7)). The high-frequency normal modes (1000 < *ħω*_*m*_ ≤ 1800 cm^−1^) can be characterized by a mean frequency *ω*_M_ and an effective electron-phonon coupling strength (or Huang-Rhys factor) *S*_M_:^21^14a
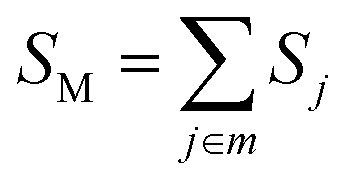
14b
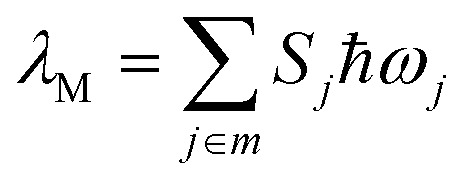
14c
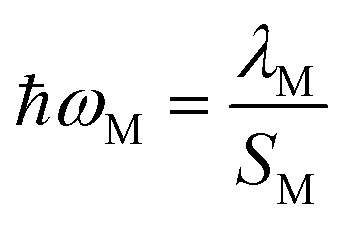


Further computational details can be found in the ESI.†

## Results and discussion

### Ground state structures and absorption energies

In general, the optimized ground state structures of 1, 3-exo, and 3-endo are in good agreement with the X-ray crystallography data (<0.05 Å and 8.5°) except for the dihedral angle between the planes of the [C^N^C] ligand and the phenyl ring of the acetylide ligand (*δ*); calculations revealed a nearly coplanar geometry (*δ* ∼ 5.7° and −0.27° for 1 and 3-exo respectively) whereas experimentally determined *δ* values are 66.1° and 54° respectively.^3*a*,5*b*^ Similarly, though DFT calculations predict a non-coplanar geometry for the ground state of 3-endo (*δ* ∼ 130°), the corresponding X-ray data is only ∼59° (see ESI† for the X-ray data and DFT results for 3-endo). In addition, the Au–C(acetylide) distance for 1 was calculated to be 1.950 Å while the corresponding distance from the crystallography data is 2.009 Å.^5*b*^ It should be noted that the Au–C(acetylide) distances reported for similar [(C_H_^N^C)Au^III^C≡CPh-4-Y] (Y is a substituent) complexes are in the range of 1.945–1.980 Å;^5*b*^ our calculated value falls within this range. It is thus possible that the discrepancies between experimental and calculated geometries are due to the crystal packing effect.

Table 2 presents the absorption energies of low-lying singlet excited states at the respective optimized S_0_ geometries of the four complexes studied herein. A full list of the TDDFT results can be found in the ESI.† In general, the calculated absorption energies are in good agreement with the corresponding experimental absorption peak maxima. Previous TD-B3LYP/CPCM calculations also suggest that the lowest absorption peak of 1 is ^1^LLCT in nature (LLCT = ligand-to-ligand charge transfer), with a calculated vertical excitation energy at *λ* = 408 nm (*f* = 0.23).^5*b*^

**Table 2:** Singlet excited state energies (*λ* in nm) and the associated oscillator strengths (*f*), together with the nature of singlet excited states of the four complexes depicted in Chart 1 at their respective optimized S_0_ geometries. *μ*^GS^(*D*) is the ground state dipole moment obtained from DFT calculations. The experimental values (*λ*_exp_ in nm) are listed in the last column

Complexes	S_*m*_	*λ*	*F*	Nature^a^	*μ* ^GS^	*λ*_exp_
1	S_1_	392	0.251	^1^LLCT	6.13	400, 380, 362
	S_2_	367	0.0519	^1^ππ*(C_H_^N^C)		
2	S_1_	401	0.2737	^1^LLCT	8.36	396, 380
	S_2_	370	0.2623	^1^ππ*(C_np_^N^C)		
3-exo	S_1_	409	0.1645	^1^ππ*(C_fl_^N^C)/^1^LLCT	8.09	428, 409
	S_2_	401	0.3078	^1^LLCT/^1^ππ*(C_fl_^N^C)		
3-endo	S_1_	426	0.0671	^1^LLCT	8.09	430, 409
	S_2_	407	0.2505	^1^ππ*(C_fl_^N^C)		

a All the singlet excited states have some metal character, but generally less than 10%.

**Fig. 2:**
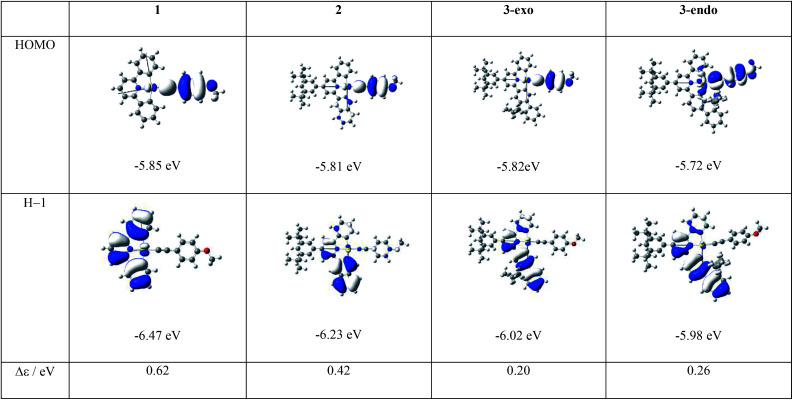
Frontier MOs of the four complexes at their respective optimized S_0_ geometries together with the HOMO/H − 1 MO splitting, Δ*ε*.

As depicted in Table 2, the most conspicuous difference among the four complexes is that, except for 3-exo, the first singlet excited state (S_1_) is a ^1^LLCT excited state, derived mainly from the HOMO → LUMO transition, ^1^[π(C≡CPh-4-OMe) → π*(C^N^C)] (Fig. 2 and ESI† for the MO surfaces). On the other hand, for 3-exo, the S_1_ state is predominantly intraligand (IL) in character (>80%); this ^1^IL excited state is derived from the H − 1 → LUMO transition and is a ^1^ππ*(C^N^C) excited state. The difference in the nature of the S_1_ excited state among the four complexes can be rationalized as follows: upon increasing the π-conjugation along the series 1, 2, 3-endo, and 3-exo, H − 1 is destabilized and the MO splitting (Δ*ε*) between HOMO and H − 1 decreases from 0.62 eV (1) to 0.26 eV (3-endo) and 0.20 eV (3-exo), (Fig. 2). This decrease in MO splitting results in a decrease in the contribution of the HOMO → LUMO transition to the S_1_ state, but a concomitant increase in percentage of the H − 1 → LUMO transition (Table S9†). As a result, the predominant contribution to the S_1_ state is mainly ^1^LLCT in character for 1, 2, and 3-endo, while for 3-exo, the S_1_ state is mainly ^1^IL in nature. This decrease in MO splitting not only affects the nature of the lowest singlet excited state, but also significantly impacts the emitting excited state, as described in a later section.

### T_1_ excited state: radiative and non-radiative decay rates

The experimental photophysical data regarding the emissions of the four gold(iii) complexes are listed in Table 3.

**Table 3:** Experimental emission maxima (*λ*_max_ nm^−1^), quantum yields (*ϕ*) and lifetimes (*τ* μs^−1^) of the four complexes measured in dichloromethane solutions at 298 K. Radiative (*k*_r_) and non-radiative (*k*_nr_) decay rates are obtained from *k*_r_ = *ϕ*/*τ* and *k*_nr_ = 1/*τ* − *k*_r_ and are tabulated in units of (×10^3^ s^−1^)

	*λ*_max_	*ϕ*	*τ*	*k*_r_	*k*_nr_
1 (ref. 3*a* and 5*b*)	474	0.0004	0.017	23.5	58 800
2 (ref. 8*a*)	562	0.09	25	3.60	36.4
3-exo (ref. 3*a*)	538	0.58	242	2.40	1.74
3-endo^a^	536	0.02	14.5	1.38	67.6

a This work, ESI.†

As depicted in Table 3, 1 has the fastest radiative and non-radiative decay rate constants, with the latter being more than 800-fold faster than that of the other three complexes. Complex 3-exo displays the slowest *k*_nr_ among the four complexes studied herein, while the associated *k*_r_ is comparable to the other two complexes with π-conjugation at the [C^N^C] cyclometalated ligand (*i.e.*, 2 and 3-endo).

**Fig. 3:**
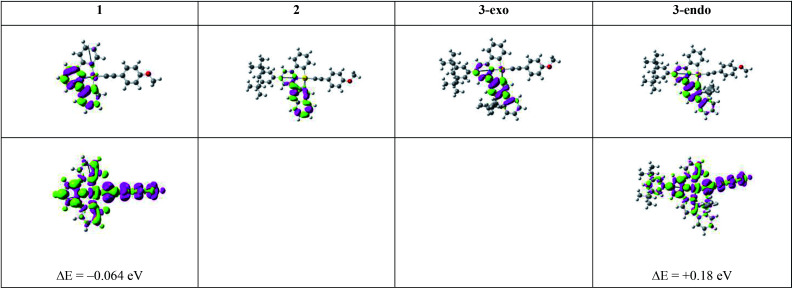
Electron difference density maps (eddms) as obtained from SS-TDDFT calculations at their respective optimized T_1_ excited state geometries for the four complexes in Chart 1 (isovalue = 0.001 a.u.). The upper row depicts the ^3^IL excited states while the bottom row presents the ^3^LLCT excited states. The ^3^IL excited state is set as the reference point, *i.e.*, Δ*E* (in eV) is the energy of the ^3^LLCT excited state relative to that of the ^3^IL excited state of a complex obtained from UDFT calculations. Colour scheme: moss green represents increased electron density; magenta represents decreased electron density.

To understand the emission properties of the four complexes depicted in Chart 1, we have employed unrestricted DFT (UDFT) to optimize their lowest triplet excited states. For 2 and 3-exo, only one triplet excited state, ^3^ππ*(C^N^C) IL excited state, was found. On the other hand, two triplet excited state minima, one ^3^IL in character and the other ^3^LLCT (^3^[π(C≡CPh-4-OMe) → π*(C^N^C)]), were found for both 1 and 3-endo. The electron difference density maps (eddms) for the calculated triplet excited states, together with the relative energy splitting between the ^3^IL and ^3^LLCT excited states for complexes 1 and 3-endo, are presented in Fig. 3.

Table 4 lists the computed 0–0 transition energies (Δ*E*_00_), vertical emission energies (Δ*E*^SS^_em_, Fig. 1), Franck–Condon factor-weighted emission energies (〈*ν̃*〉_fcf_), and radiative decay rate constants of the optimized T_1_ excited states of the four gold(iii) complexes studied herein.

**Table 4:** Computed 0–0 transition energy (Δ*E*_00_ in nm), vertical emission energy (Δ*E*^SS^_em_ in nm), Franck–Condon-factor weighted emission energy (〈*ν̃*〉_fcf_ in nm), and radiative decay rate constants (*k*_r_× 10^3^ s^−1^) for the four gold(iii) complexes^a^

		Δ*E*_00_	Δ*E*^SS^_em_		〈*ν̃*〉_fcf_	*k*_r_ b
			SCF	SS-TDDFT		
1	^3^IL	484	534	612	555	6.12 (8.28)
	^3^LLCT	492	586	1832	—c	0.018
2	^3^IL	541	593	704	618	0.148 (0.219)
3-exo	^3^IL	554	610	698	621	0.544 (0.772)
3-endo	^3^IL	550	604	691	612	0.353 (0.507)
	^3^LLCT	510	601	1570	—c	0.047

a Δ*E*^SS^_em_ is obtained from two different methods: (1) in the SCF method, it is the energy difference between the T_1_ excited state calculated with equilibrium solvation at the UDFT level and the S_0_ ground state with non-equilibrium solvation with the T_1_ excited state electron density using DFT; (2) in the SS-TDDFT method, it is the pole of the T_1_ excited state from a SS-TDDFT calculation with PCM correction; 〈*ν̃*〉_fcf_ is obtained from eqn (7) using the emission spectrum generated from a Franck–Condon calculation implemented in G09 (for details, see above and ESI†); *k*_r_ is the radiative decay rate constant obtained by considering only the lowest singlet excited state(s) that can have effective SOC with the T_1_ excited state (see ESI† for further computational details).

b The value outside the parentheses corresponds to the radiative decay rate constant obtained using SS-TDDFT Δ*E*^SS^_em_ while that inside the parentheses corresponds to that obtained using 〈*ν̃*〉_fcf_.

c FC simulated spectrum is unreliable; and therefore 〈*ν̃*〉_fcf_ cannot be determined in such a case.

#### (i) Emission energies

With the exception of 2, there is generally a close correlation between the experimental solution emission maxima (*λ*_max_) at room temperature and the calculated Δ*E*_00_ of the ^3^IL excited states of the gold(iii) complexes in Chart 1. This suggests that for complexes 1, 3-exo, and 3-endo, the emission maximum may correspond to the 0–0 transition of ^3^IL → S_0_. The experimental emission maximum of 2 is at a lower energy than that of 3-exo (Table 3). For related platinum(ii) [C^N^C] cyclometalated complexes, the one with a naphthalene moiety at the [C^N^C] ligand displays a *higher energy* emission peak than the one with a fluorene unit (*e.g.*, complexes 7 and 8 in ref. 9) and the emitting triplet excited state is assigned as having a mixed ^3^IL/^3^MLCT character (MLCT = metal-to-ligand charge transfer).^9^ Our present theoretical analysis is in accordance with these findings on the platinum(ii) [C^N^C] cyclometalated complexes: Δ*E*_00_ of the gold(iii) complexes is in the order 1 > 2 > 3-endo ∼ 3-exo. This trend is a manifestation of the increase in π-conjugation at the [C^N^C] cyclometalated ligand when one goes from 1 to 2 to 3-endo and 3-exo. Increasing π-conjugation destabilizes the π(C^N^C) orbital, (see also Fig. 2), thereby decreasing the MO splitting between π(C^N^C) and π*(C^N^C) orbitals and leading to a red shift in emission energy of the ^3^IL excited state from 1 to 2 to 3-endo and 3-exo. The fact that the experimental emission maximum of 2 is lower in energy than those of 3-exo and 3-endo may reflect that the emission peak maximum of 2 may not correspond to the 0–0 transition; it may suggest that the structural distortion between the T_1_ and S_0_ states of 2 is larger than that of 3-exo and 3-endo (*vide infra*).

#### (ii) Radiative decay rate constants

Table 4 presents the radiative decay rate constants calculated for each of the T_1_ excited states of the four complexes. Although the *k*_r_ values of the ^3^IL excited states are slightly underestimated by a factor of ∼2.7–3.1, they are consistent with the experimental *k*_r_ values except in the case of 2 (compare Tables 3 with 4). The calculations indicate that 2 should have the slowest radiative decay rate constant, which is not supported by the photophysical data recorded at room temperature (Table 3). However, it was reported that the emission lifetime of 2 increases from 25 μs at room temperature to 2285 μs in a glassy medium at 77 K.^8*a*^ Assuming that this lifetime corresponds to the radiative lifetime, *k*_r_ would be estimated to have a maximum value of ∼438 s^−1^. This is close to our theoretical results, *i.e.*, the ^3^IL excited state of 2 should have the slowest radiative decay rate constant among the four gold(iii) complexes (as a reference, the *k*_r_ estimated in the same way as that of 2 at 77 K would be 7.30 × 10^3^, 3.55 × 10^3^, and 2.46 × 10^3^ s^−1^ for 1, 3-exo, and 3-endo respectively).^3*a*,5*b*^ It is conceivable that the emission of 2 at 298 K and 77 K originated from different excited states. However, no other triplet excited state minimum was found for complex 2 using the present DFT/TDDFT method.

#### (iii) Non-radiative decay rate constants

Table 5 lists the calculated results related to the non-radiative decay rate constants. First, let us consider the ^3^IL excited states of the four gold(iii) complexes. As depicted in Table 5, the Huang–Rhys factors (*S*_M_) are in the order: 1 > 2 > 3-exo ∼ 3-endo. This trend is in line with the S_0_ to T_1_ structural distortion of the following organic molecules in the order: benzene > naphthalene > carbazole (carbazole is isoelectronic to fluorene).^22^ These two trends are similar because the ^3^IL excited states of these four gold(iii) complexes are mainly localized on the phenyl, naphthalenyl, and fluorenyl moieties, respectively (Fig. 3). As the Huang–Rhys factor serves to quantify the structural distortion between the emitting triplet excited state and the ground state, the smallest values of *S*_M_ for 3-exo and 3-endo reveal that the fluorene unit at the [C^N^C] cyclometalated ligand imparts the greatest rigidity to the complex. In other words, the rigidity of the organic moiety at the pincer-type cyclometalated ligand could qualitatively account for the experimental results that 1 has the fastest non-radiative decay rate constant and 3-exo the slowest.

**Table 5:** Effective Huang–Rhys factors (*S*_M_) for the high-frequency mode, intramolecular (*λ*_v_) and solvent (*λ*_s_) reorganization energies (in cm^−1^), dipole moments of the T_1_ excited state (*μ*^T1^/*D*), 〈T_1_|*H*_SOC_|S_0_〉^2^ (in cm^−2^), Franck–Condon Factors (FCF), and non-radiative decay rate constants (*k*_nr_ × 10^3^ s^−1^) for the four complexes studied herein

		*S*_M_ a	*λ*_v_ b		*λ*_s_	*μ* ^T1^	〈T_1_|*H*_SOC_|S_0_〉^2^	FCF^e^	*k*_nr_
			SS	FC					
1	^3^IL	1.75	2889	2920	21.2	6.23	943	2.66	2.508
	^3^LLCT	0.11^c^	2090	n.a.	1980	16.3	1757	989^d^	1738
2	^3^IL	1.47	2622	2618	62.72	8.36	148	151	22.35
3-exo	^3^IL	1.29	2392	2408	75.74	7.21	323	9.74	3.146
3-endo	^3^IL	1.27	2388	2439	83.24	6.29	173	7.24	1.253
	^3^LLCT	0.22^c^	2051	n.a.	1812	18.5	1664	1130^d^	1880

a *S*_M_ corresponds to the effective Huang–Rhys factor of the high-frequency (hf) modes in the range 1000 ≤ *ω*_*m*_ ≤ 1800 cm^−1^ when the T_1_ excited state is ^3^IL.

b The intramolecular reorganization energy *λ*_v_ was obtained in two different ways: state specific (SS; eqn (3)) and Franck–Condon (FC; eqn (13)).

c *S*_M_ = *S*_C_, *i.e.*, the Huang–Rhys factor of the C≡C stretching mode, *ω*_C≡C_ (see ESI† for details).

d Estimated under the assumption that the Huang–Rhys factors of the ^3^LLCT → S_0_ transition are the same as those of the ^3^IL → S_0_ of the same complex, together with the Huang–Rhys factor of the C≡C stretching normal mode.

e The term 2π/*ħ* is absorbed into the FCF.

Besides, the magnitude of the SOC matrix element between the ^3^IL excited state and S_0_ ground state follows the order: 1 > 3-exo > 3-endo > 2. At their respective optimized ^3^IL excited states, the metal contributions (expressed as *c*_d_^2^) to the H − 1 (HOMO for 3-exo and 3-endo), at their optimized T_1_ geometries, are 4.18 (1), 0.36 (2), 1.94 (3-exo), and 1.07 (3-endo), respectively. As SOC is mainly brought about by the gold(iii) ion, the larger the coefficient of Au(d) in the H − 1/HOMO, the larger should be the SOC matrix element, 〈^3^IL|*H*_SOC_|S_0_〉^2^. The Au(d) character in the H − 1/HOMO of the gold(iii) complexes studied herein is related to the nature of the HOMO of the C-deprotonated moiety in the [C^N^C] ligand. For complex 2, the H − 1 is mainly localized on the *long* molecular axis of the naphthalene fragment (Fig. 2), thus rendering the [C_np_^N^C] ligand to have little interaction with the gold(iii) ion and therefore, the smallest *c*_d_ in the H − 1 orbital of 2. On the other hand, the corresponding orbital of complex 3-exo is along the *short* molecular axis of the fluorene fragment, thus the [C_fl_^N^C] ligand could have a stronger interaction with the gold(iii) ion, and hence, a larger *c*_d_ in the HOMOs of complexes 3-exo and 3-endo.

**Fig. 4:**
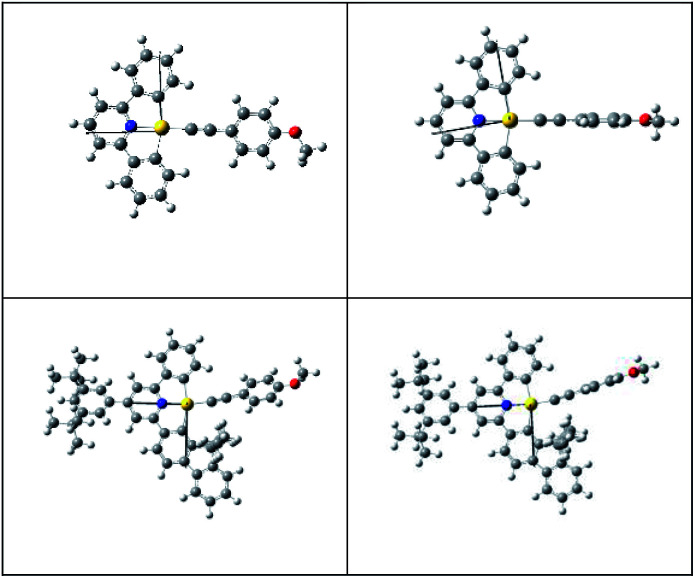
Optimized structures of the S_0_ (left) and ^3^LLCT excited states for 1 (top) and 3-endo (bottom).

Although both the effective Huang–Rhys factor *S*_M_ and the SOC between the T_1_ and S_0_ states are largest for 1, the calculated non-radiative decay rate constant *k*_nr_ for the ^3^IL → S_0_ transition is *smaller* than that of 3-exo, a result contrary to the order of experimental *k*_nr_ values; *k*_nr_(calc): 2 > 3-exo > 1; *k*_nr_(expt): 1 ≫ 2 > 3-exo. This is because 1 has a much larger energy gap between the ^3^IL and S_0_ states than the other three gold(iii) complexes (Table 4), making the energy gap effect play a dominant role in determining the *k*_nr_ (^3^IL → S_0_) of 1. Similarly, the calculated non-radiative decay rate constant for 3-endo is ∼1.25 × 10^3^ s^−1^, which is also *smaller* than that of 3-exo, and is inconsistent with the experimental data (compare Tables 3 and 5). For these two complexes, 1 and 3-endo, an additional triplet excited state minimum was found (Fig. 3). This triplet excited state, as observed from the eddms in Fig. 3, is best characterized to be a ^3^LLCT, ^3^[π(C≡CPh-4-OMe) → π*(C^N^C)], excited state. This ^3^LLCT excited state displays a large amplitude motion along the dihedral angle between the [C^N^C] plane and the arylacetylide plane (*δ*): from ∼−4.132° (S_0_) to −88.739° (^3^LLCT) for 1 and from 130.381° (S_0_) to 92.352° (^3^LLCT) for 3-endo (see Fig. 4 for the optimized structures of the S_0_ and ^3^LLCT excited states for complexes 1 and 3-endo). Because of this large amplitude motion, we refrained from performing a Franck–Condon calculation on the ^3^LLCT → S_0_ transition, as we have performed for that of the ^3^IL → S_0_. This is because, for the Franck–Condon calculation implemented in G09, the normal modes are represented in Cartesian coordinates. Cartesian coordinates are inadequate to describe large amplitude motions, such as torsions, as this could lead to artificial bond breaking and bond forming at its extreme.^23^ For instance, due to the rotation of the phenyl group at the arylacetylide ligand relative to the [C^N^C] plane, the C–H bonds on the phenyl ring of the arylacetylide ligand would be artificially broken if Cartesian coordinates were used to describe the normal modes. This could result in erroneously large Huang–Rhys factors for the C–H stretching modes. However, in reality, there is no C–H bond breaking when one goes from the ^3^LLCT to the S_0_ state. Moreover, such fictitious bond breaking and bond forming will lead to a diffuse Duschinsky matrix, which could lead to an incorrect interpretation of the fast non-radiative decay rate constant due to a large Duschinsky effect.

Nevertheless, the C≡C stretching normal mode is decoupled from the other normal modes, as reflected by the Duschinsky matrix elements of the ^3^LLCT → S_0_ transition; *ω*_C≡C_ is the only normal mode that has the diagonal matrix element equal to 1. Therefore, we estimated the non-radiative decay rate constants of the ^3^LLCT excited state by replacing all the Huang–Rhys factors (*S*_*j*_) of the ^3^LLCT → S_0_ transition with those of the ^3^IL → S_0_ transition, but keeping the Huang–Rhys factor of the C≡C stretching normal mode from a Franck–Condon calculation of the ^3^LLCT → S_0_ transition. Such an assumption is based on the fact that both the ^3^LLCT and ^3^IL excited states of the gold(iii) complexes involve changes in electron density at the [C^N^C] ligand.

From Table 5, several points concerning the ^3^LLCT excited states of 1 and 3-endo are noted: (a) The solvent reorganization energy (*λ*_S_) of ^3^LLCT is much larger than that of ^3^IL. This is attributed to the dipole moment of the ^3^LLCT being much larger than that of ^3^IL and the ground states (see Table 5 for the excited state dipole moments (*μ*^T1^) and Table 2 for the ground state dipole moments (*μ*^GS^)). In the framework of the SS approach, solvent reorganization energy is proportional to the square of the difference in dipole moments between the T_1_ excited state and the S_0_ ground state, *i.e.*, *λ*_s_ ∝ (*μ*^T1^ − *μ*^GS^)^2^.^20^ Therefore, this large solvent reorganization effectively leads to a decrease in the energy gap between the ^3^LLCT and the S_0_ potential energy surfaces (PESs) at the equilibrium geometry of the ^3^LLCT excited state. Thus, fewer quanta of the high-frequency vibrational mode (*n*_M_) are needed (see eqn (12e)) and the activation energy (the temperature-dependent term in the last exponential of eqn (11)) is smaller as this energy term is inversely proportional to the solvent reorganization energy; (b) the square of the *H*_SOC_ matrix element between the ^3^LLCT excited state and the S_0_ ground state is larger than that between the ^3^IL excited state and the ground state (Table 5).

**Fig. 5:**
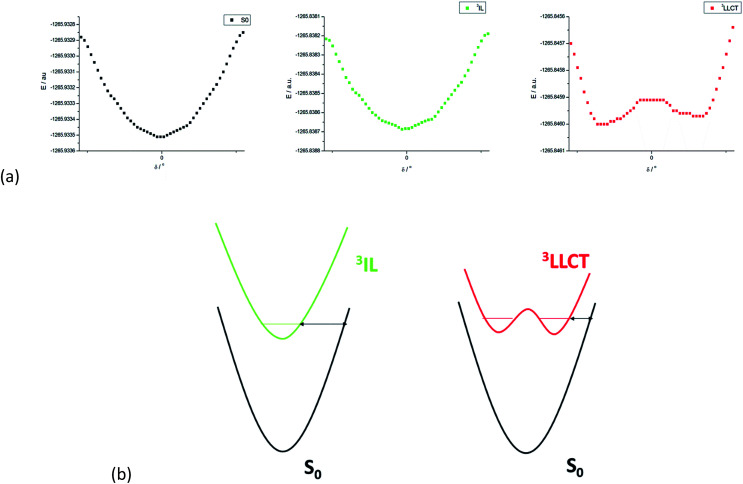
(a) Potential energy surface (PES) of 1 along the torsion coordinate (*δ*) for the S_0_ state (left), ^3^IL excited state (middle), and ^3^LLCT excited state (right). (b) The left-hand PESs depict the case when both PESs in a transition are harmonic and the right-hand PESs represent the case when the emitting excited state has a double minimum PES; the double arrow indicates the “barrier width” and it is smaller for the right-hand case than the left-hand case.

The non-radiative decay rate constants thus estimated for the ^3^LLCT excited states of 1 and 3-endo are 1.738 × 10^6^ and 1.880 × 10^6^ s^−1^, respectively, more than 690-fold and over 1500-fold larger than those of their respective ^3^IL excited states. These non-radiative decay rate constants may still be underestimated since the structural change associated with the torsional motion between the [C^N^C] and arylacetylide ligands has not been included in the Franck–Condon factor (FCF) calculation of the ^3^LLCT → S_0_ transition. (We have used the Huang–Rhys factor of the ^3^IL → S_0_ transition where there is no such large amplitude torsion.) We have undertaken a rigid scan along the torsional coordinate (*δ*) for 1. Fig. 5a displays the PESs along the torsional coordinate *δ* for the ground state, ^3^IL excited state, and ^3^LLCT excited state of complex 1. The potential energy minimum is roughly harmonic for both the ground state and the ^3^IL excited state but anharmonic for ^3^LLCT excited state. As the ^3^LLCT excited state has a double minimum potential while the ground state is approximately harmonic, the Franck–Condon factor (FCF) between ^3^LLCT and S_0_ is expected to be larger than that between the ^3^IL and S_0_ states, where both PESs are harmonic along the torsion coordinate *δ*. This may be rationalized as illustrated in Fig. 5b. The “barrier width” (indicated by the double arrow in Fig. 5b), being qualitatively related to the FCF in an inverse manner, is smaller for a potential energy surface with a double minimum potential (as in ^3^LLCT excited state) than that with a harmonic PES (as in ^3^IL excited state; Fig. 5b).^24^ Thus, the non-radiative decay rate of the ^3^LLCT → S_0_ transition should be further enhanced due to the increase in the FCF brought about by the torsional motion. In addition, there would be a strong thermal quenching of phosphorescence because thermal excitation of the torsional normal mode in the ^3^LLCT excited state would decrease the “barrier width”, leading to a significant increase in the FCFs, and, hence, a further enhancement of the non-radiative decay rate.

If one supposes that the torsional motion increases the FCF of the ^3^LLCT → S_0_ transition by a factor of ∼10, the values of *k*_nr_ for 1 and 3-endo for this transition would be ∼1.7 × 10^7^ and ∼1.9 × 10^7^ s^−1^, respectively. We may then re-estimate the non-radiative decay rate constants by taking into consideration both the ^3^LLCT and ^3^IL excited states with Boltzmann populations. As ^3^LLCT is calculated to be ∼500 cm^−1^*below*^3^IL for complex 1, the re-estimated non-radiative decay rate constant for complex 1 at room temperature is comparable to the experimental value (*k*_nr_(calc) ∼ 1.6 × 10^7^ s^−1^ and *k*_nr_(expt) ∼ 5.9 × 10^7^ s^−1^). In other words, the major deactivating channel for the emissive excited state of 1 is not ^3^dd or ^3^LMCT, as is usually ascribed to efficient non-radiative decay for luminescent transition metal complexes, but ^3^LLCT due to a large SOC, a large solvent reorganization energy, and the non-planar torsional motion between the [C^N^C] and arylacetylide ligands. For 3-endo, the ^3^LLCT excited state is calculated to be ∼1400 cm^−1^*above* that of the ^3^IL state. Therefore, the re-estimated *k*_nr_ becomes ∼1.5 × 10^4^ s^−1^, which is in good agreement with the values derived from the experimental measurements in solutions at 298 K (*k*_nr_(expt) ∼6.8 × 10^4^ s^−1^).

**Fig. 6:**
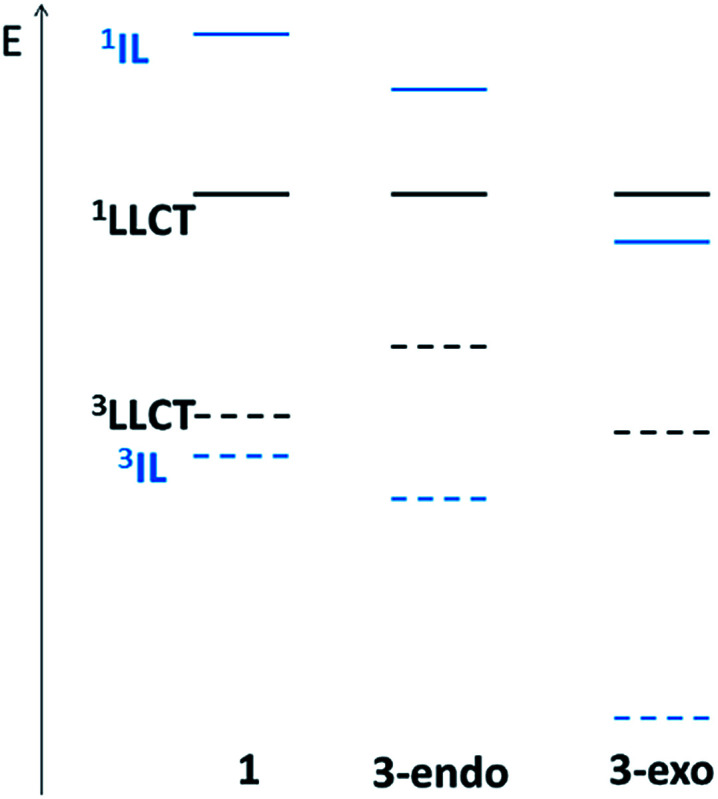
Schematic representation of the relative ^3^LLCT–^1^LLCT and ^3^IL–^1^IL splittings for 1 (left), 3-endo (middle), and 3-exo (right). The solid curve corresponds to a singlet excited state while the dashed line a triplet excited state. The colours black and blue represent the LLCT and IL excited states, respectively.

Based on the above analyses on non-radiative decay rate constants, it is the presence of the close-lying ^3^LLCT excited state that contributes to the very fast non-radiative decay rate. The relative order of the ^3^LLCT and ^3^IL excited states would thus be important in determining the phosphorescence efficiency. In the present series of gold(iii) complexes, this relative order can be understood from the relative energies of the π(C^N^C) and π(C≡CPh-4-OMe) MOs. As the LLCT excited state is a charge transfer excited state, while the IL excited state is localized, the singlet–triplet splitting of LLCT excited states (*E*(^1^LLCT)–*E*(^3^LLCT)) would be smaller than that of IL excited states (*E*(^1^IL)–*E*(^3^IL)). In the case of 1, due to the large orbital energy difference (Δ*ε*) between the π(C_H_^N^C) and π(C≡CPh-4-OMe) MOs (Fig. 2), the ^1^IL excited state is much higher in energy than that of the ^1^LLCT excited state. Thus, the splitting of the ^3^IL and ^3^LLCT states is the smallest (see Fig. 6 for a schematic illustration). For 3-endo, as the corresponding Δ*ε* is smaller than that of 1, the ^1^LLCT is only slightly lower in energy than the ^1^IL excited state such that the ^3^IL–^3^LLCT energy gap widens. For 3-exo, as the lowest singlet excited state is predominantly IL in character, the ^3^IL–^3^LLCT energy gap is even wider. In fact, we have not been able to locate a T_1_ minimum corresponding to a ^3^LLCT excited state (Fig. 6).

Based on the above rationale, it is speculated that the ^3^IL–^3^LLCT gap of 2 should fall between that of 1 and 3-endo, as deduced from the relative order of the π(C_np_^N^C) and π(C≡CPh-4-OMe) MOs depicted in Fig. 2. Indeed, an energy minimum of a ^3^LLCT excited state was located in the course of LR-TDDFT optimization; subsequent SS-TDDFT calculation at this geometry showed that this ^3^LLCT excited state is *lower-lying* than the ^3^IL one. However, global hybrid density functionals, (*e.g.*, PBE0, a functional that we have employed in the present work) generally underestimate the energy of charge transfer excited states within the TDDFT framework. Thus, we performed UDFT optimization starting from these TDDFT-optimized structures (which have a stable wavefunction) to see if there is a ^3^LLCT excited state minimum. Unfortunately, UDFT optimization starting from the TDDFT-optimized ^3^LLCT excited state went back to the ^3^IL excited state. It is likely that this ^3^LLCT excited state is metastable and exhibits vibronic coupling with other close-lying excited states.

## Conclusions

We have carried out a detailed theoretical study on four gold(iii) [C^N^C] cyclometalated complexes with different extents of π-conjugation. It is commonly prescribed that a rigid ligand in a transition metal complex can minimize structural distortion between the emitting triplet excited state and the ground state, thereby decreasing the non-radiative decay rate. Franck–Condon analyses on the ^3^ππ*(C^N^C) IL → S_0_ transitions of the four gold(iii) complexes confirmed that an increase in π-conjugation at the [C^N^C] cyclometalated ligand results in a more rigid transition metal complex, as reflected by the effective Huang–Rhys factor, *S*_M_: 1 > 2 > 3-exo and 3-endo. Although this trend correlates with the experimentally determined non-radiative rate constants, 1 ≫ 2 > 3-exo, the calculated *k*_nr_ of the ^3^IL → S_0_ transition is inconsistent with the experimental data if one also takes into consideration the ^3^IL–S_0_ energy gap. DFT/TDDFT calculations reveal that there is an additional triplet excited state minimum, ^3^[π(C≡CPh-4-OMe) → π*(C^N^C)] LLCT, for complexes 1 and 3-endo, but not for 3-exo. It was found that the non-radiative decay rate constant for this ^3^LLCT → S_0_ transition exceeds 10^7^ s^−1^, which is more than three orders of magnitude faster than the *k*_nr_ for the ^3^IL → S_0_ transition. More importantly, if the relative splitting between the ^3^LLCT and ^3^IL excited states was included in estimating the *k*_nr_ of complexes 1 and 3-endo, the calculated and experimental *k*_nr_ are in *quantitative* agreement. Based on the analysis of the relative order of π(C^N^C) and π(C≡CPh-4-OMe) MOs, one could rationalize why complexes 1 and 3-endo, but not 3-exo, have low-lying ^3^LLCT excited states. Our present analysis highlights the importance of the relative order of the frontier MOs of the coordinating ligands in multi-chromophoric transition metal complexes in designing strongly luminescent transition metal complexes. It also challenges the presumption that the low phosphorescence efficiency of transition metal complexes is due to the close proximity of the dd ligand-field state to the emitting triplet excited state.

## Appendix

**Table:** 

List of definitions, abbreviations, and symbols	
Abbreviation	Definition
IL	Intraligand
LLCT	Ligand-to-ligand charge transfer
LMCT	Ligand-to-metal charge transfer
MLCT	Metal-to-ligand charge transfer
SOC	Spin–orbit coupling
LR	Linear response
SS	State-specific
EQ	Equilibrium
NEQ	Non-equilibrium
PCM	Polarizable continuum model
FCF	Franck–Condon factor
PES	Potential energy surface
Expt	Experimental
Calc	Calculated
eddm	Electron difference density map
*μ* ^GS^	Dipole moment of the ground state
*μ* ^T1^	Dipole moment of the T_1_ excited state
*c*_d_	Coefficient of Au(d-orbital)
*Q* ^GS^_0_	Optimized *ground* state (GS) geometry
*Q* ^ES^_0_	Optimized excited state (ES) geometry
Δ*E*^SS^_em_	Emission energy evaluated within the state-specific (SS) approach; eqn (1), Fig. 1
*E* ^ES^_EQ_(*Q*^ES^_0_)	Energy of the *excited* state (ES) with *equilibrium* (EQ) solvation at the optimized excited state geometry, Fig. 1
*E* ^GS^_NEQ_(*Q*^ES^_0_)	Energy of the *ground* state (GS) with *non-equilibrium* (NEQ) solvation at the optimized excited state geometry, Fig. 1
*E* ^GS^_EQ_(*Q*^ES^_0_)	Energy of the *ground* state (GS) with *equilibrium* (EQ) solvation at the optimized excited state geometry, Fig. 1
*E* ^GS^_EQ_(*Q*^GS^_0_)	Energy of the *ground* state (GS) with *equilibrium* (EQ) solvation at the optimized ground state geometry, Fig. 1
*λ*_s_	Solvent reorganization energy; eqn (2)
*λ* ^SS^_V_	Intramolecular reorganization energy evaluated within the state-specific (SS) approach; eqn (3)
*λ* ^FC^_V_	Intramolecular reorganization energy obtained from Franck–Condon (FC) calculation; eqn (13)
*υ*′	Vibrational quantum number of the first triplet (T_1_) excited state
*υ*′′	Vibrational quantum number of the ground state (S_0_)
*χ*_*υ*′_	Vibrational wavefunction of the T_1_ excited state
*χ*_*υ*′′_	Vibrational wavefunction of the ground state
*η*	Solvent refractive index
***M***_T_^*α*^(*Q*)	Transition dipole moment of the T_1_^*α*^ → S_0_ transition at geometry, Q
***M***_T_^***α***^(*Q*^T1^_0_)	Transition dipole moment of the T_1_^*α*^ → S_0_ transition evaluated at the optimized T_1_ geometry, *Q*^T1^_0_; eqn (9)
***M***_S_*m*_, *j*_(*Q*^T1^_0_)	*j*-axis projection of the transition dipole moment of the S_*m*_ → S_0_ transition evaluated at the optimized T_1_ geometry, *Q*^T1^_0_; *j* = *x*, *y*, or *z*
*I*(*ν̃*)	Emission intensity at (*ν̃*)
〈*ν̃*〉_fcf_	Franck–Condon factor weighted emission energy; eqn (7)
*H*_*SOC*_	Spin–orbit coupling operator
Δ*E*_00_	Zero-point energy difference between the emitting state and the ground state
*ħω*_j_	Vibrational frequency of the *j*^th^ normal mode (in cm^−1^)
Δ*Q*_*j*_	Equilibrium displacement of the *j*^th^ normal mode
*S*_*j*_	Huang-Rhys factor of the *j*^th^ normal mode; eqn (12c)
*ħω*_lf_	Vibrational frequency of the low-frequency (lf) normal modes: *ħω*_lf_ ≤ 1000 cm^−1^
*λ*_lf_	Intramolecular reorganization energy contributed by the low-frequency (lf) normal modes; eqn (12b)
*ħω*_*m*_	Vibrational frequency of the high-frequency (hf) normal modes in the range: 1000 < *ħω*_*m*_ ≤ 1800 cm^−1^
*ħω*_M_	Mean frequency of the high-frequency normal modes, *ω*_*m*_; eqn (14c)
*λ*_M_	Intramolecular reorganization energy contributed by the high-frequency normal modes *ω*_*m*_; eqn (14b)
*S*_M_	Effective electron-phonon coupling strength or Huang-Rhys factor of the effective normal mode, *ω*_M_; eqn (12d) and (14a)
*n*_M_	Number of vibrational quanta of *ħω*_M_; eqn (12e)

## Supplementary Material

SC-006-C4SC03697B-s001

SC-006-C4SC03697B-s002
