# Supplementary material for: Theoretical studies on the photophysical properties of luminescent pincer gold(iii) arylacetylide complexes: the role of π-conjugation at the C-deprotonated [C^N^C] ligand
Source: Chem Sci. 2015 Mar 10;6(5):3026–37. doi: 10.1039/c4sc03697b (PMC5795471; doi:10.1039/c4sc03697b)
Supplement: SC-006-C4SC03697B-s001 [file SC-006-C4SC03697B-s001.pdf]

Electronic Supplementary Material (ESI) for Chemical Science.
This journal is © The Royal Society of Chemistry 2015

Electronic Supplementary Information for

Theoretical Studies on the Photophysical Properties of Luminescent Pincer Type Gold(III) Arylacetylide Complexes: The Role of π-Conjugation at the Tridentate C-deprotonated Cyclometalated [C^N^C] Ligand.

Glenna So Ming Tong, Kaai Tung Chan, Xiaoyong Chang, and Chi-Ming Che**

State Key Laboratory of Synthetic Chemistry, Institute of Molecular Functional Materials, Department of Chemistry, The University of Hong Kong, Pokfulam Road, Hong Kong (SAR China).

Contents

1. Experimental Details.
    a. Synthesis
    b. Characterizations
    c. X-ray data, and
    d. Spectroscopic properties
2. Computational Details
    a. State specific calculation procedures
    b. Radiative decay rate calculations
    c. Non-radiative decay rate calculations
    d. Further references
3. Further calculation results
    a. TDDFT results at the optimized S_0 geometries
    b. Radiative decay rate calculations
    c. Non-radiative decay rate calculations
    d. Justification of using PBE0 in predicting the relative energies of ^{3}IL and ^{3}LLCT excited states
4. Coordinates for the optimized S_0, ^{3}IL, and ^{3}LLCT excited states

Experimental Section.

Synthesis and characterization of complex 3-endo. Complex **3-endo** was synthesized according to Scheme S1. It followed the same procedure as reported in our previous paper.[1] Except that in the procedure of (e), the mercurated product was isolated by column chromatography on SiO_2 using hexane: CH_2Cl_2 = 2:1 (R_f = 0.40) as the eluent. The final product was isolated as yellow powder. Yield: 54 mg (81.7 %). X-ray crystal structure has been determined.[2]

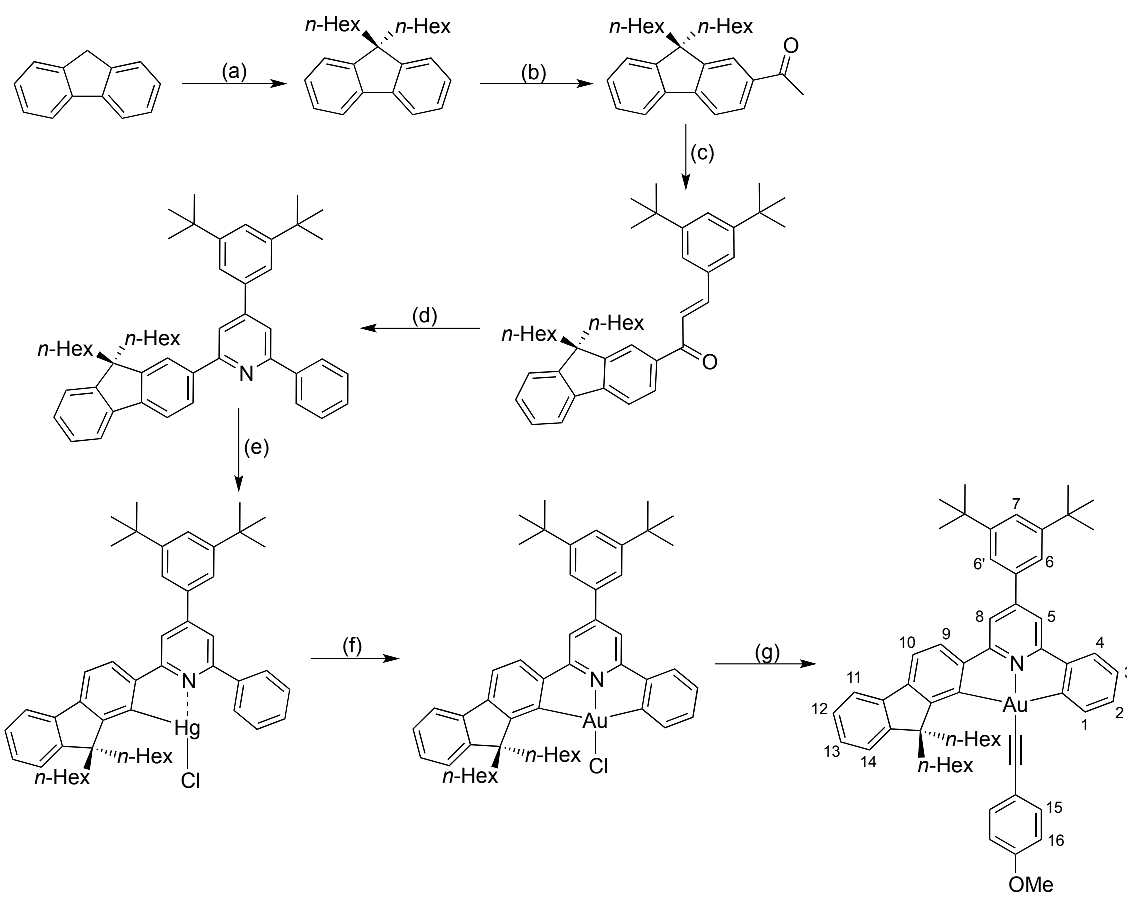

Scheme S1. Synthetic scheme for complex **3-endo**. (a) n-bromohexane, KO*t*Bu, THF, reflux. (b) $AlCl_3$, acetic anhydride, CS_2, 40°C. (c) 3,5-di-tert-butylbenzaldehyde, NaOH, MeOH, 60°C. (d) 1-(2-oxo-2-phenylethyl)pyridinium iodide, NH_4OAc, acetic acid, methanol, reflux. (e) $Hg(OAc)_2$, ethanol, reflux 48 hrs; LiCl, reflux 2 hrs. (f) $KAuCl_4$, acetonitrile, reflux 48 hrs. (g) CuI, 1-ethynyl-4-methoxybenzene, CH_2Cl_2 / NEt_3, under N_2 stir 3h.

3-endo: MS (+FAB) *m/z* (%): 1002.8 (100) [M+], 868.6 (26), 784.5 (21). ^{1}H NMR (400 MHz, [d$_6$]DMSO): δ 8.25 (d, 1H, *J* = 8.21 Hz, H^4), 8.21 (s, 1H, H^5), 8.20 (d, 1H, *J* = 8.01 Hz, H^9), 8.14 (d, 1H, *J* = 8.10 Hz, H^1), 8.13 (s, 1H, H^8),7.82–7.84 (m, 2H, H^{10} and H^{11}), 7.76 (s, 2H,H^6 and $H^{6'}$), 7.60 (s, 1H, H^7), 7.51 (d, 2H, *J* = 8.68 Hz, H^{15}), 7.47 (t, 1H, *J* = 7.88 Hz, H^3), 7.39–7.41 (m, 1H, H^{14}), 7.37 (t, 1H, *J* = 8.20 Hz, H^2), 7.30–7.33 (m, 2H, H^{12} and H^{13}), 7.01 (d, 2H, *J* = 8.74 Hz, H^{16}), 3.80 (s, 3H), 3.49-3.51 (m, 2H), 1.91-1.95 (m, 2H), 1.40 (s, 18H), 0.96–0.99 (m, 12H), 0.62 (t, 6H, *J* = 6.47 Hz), 0.42–0.47 (m, 4H). Calcd for $C_{59}H_{66}AuNO$: C, 70.71; H, 6.64; N, 1.40; found: C, 70.31; H, 6.51; N, 1.45.

Table S1. Crystal data of **3-endo**

Complex	**3-endo**
Empirical formula	$C_{65}H_{80}AuNO$
Formula weight	1088.26
Temperature/K	100
Crystal system	triclinic
Space group	P-1
a/Å	11.6132(9)
b/Å	14.7425(11)
c/Å	16.9669(13)
α/°	87.393(3)
β/°	71.112(3)
γ/°	80.062(3)
Volume/Å^3	2707.0(4)
Z	2
ρ_{calc}g/cm^3	1.335
μ/mm^{-1}	5.414
F(000)	1128
Crystal size/mm^3	0.06 × 0.06 × 0.02
Radiation	CuKα (λ = 1.54178)
2θ range for data collection/°	5.506 to 133.47
Index ranges	-13 ≤ h ≤ 13, -10 ≤ k ≤ 17, -19 ≤ l ≤ 20
Reflections collected	86638
Independent reflections	9371 [R_{int} = 0.0774, R_{sigma} = 0.0428]
Data/restraints/parameters	9371/70/650
Goodness-of-fit on F^2	1.046
Final R indexes [I>=2σ (I)]	R_1 = 0.0398, wR_2 = 0.1010
Final R indexes [all data]	R_1 = 0.0423, wR_2 = 0.1043
Largest diff. peak/hole / e Å^{-3}	1.58/-2.79

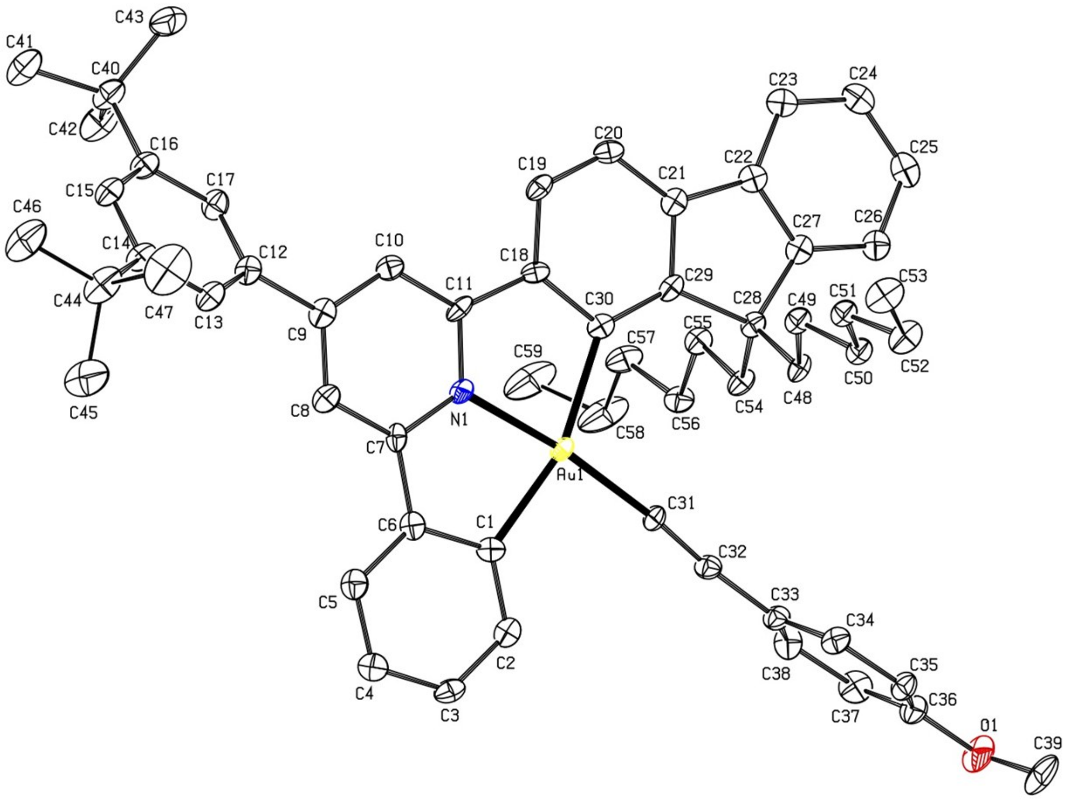

Figure S1. ORTEP diagram of **3-endo** with atomic numbering. All hydrogen atoms and solvent molecules are omitted for clarity. Thermal ellipsoids are drawn at 50 % probability level.

Table S2. Selected bond lengths and angles of **3-endo**

**3-endo**	
Bond lengths (Å)	
Au1-C1	2.063(6)
Au1-C30	2.130(5)
Au1-C31	1.947(5)
Au1-N1	1.987(3)
C31-C32	1.221(6)
C32-C33	1.443(6)
Bond angles (deg)	
C1-Au1-N1	81.1(2)
C30-Au1-N1	80.9(1)
C30-Au1-C1	162.0(2)
C31-Au1-N1	172.8(2)
Au1-C31-C32	174.2(4)
C31-C32-C33	174.0(5)

Table S3. Photophysical data of complex **3-endo**.

Complex	Medium [Temp]	UV/Vis absorption λ_{max} [nm] (ε [$mol^{-1}dm^{3}$ cm^{-1}])	Emission		
			λ_{max} [nm]	τ [µs]	ϕ_{em}
**3-endo**	DCM (298 K)	270 (56500), 305 (38500), 323 (42100), 337 (39700), 387 (8670), 409 (12100), 430 (12200)	536, 574	14.5	0.02
	EtOH : MeOH = 4 : 1 (77 K)	-	530, 576, 614 (sh)	406.5	-

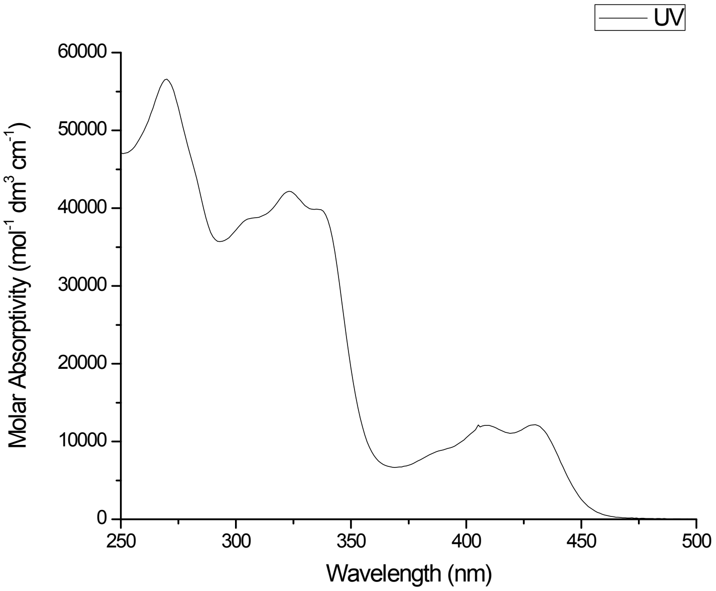

Figure S2. Electronic absorption spectrum of **3-endo** in dichloromethane (DCM) at room temperature at 2×10^{-5} mol dm^{-3}.

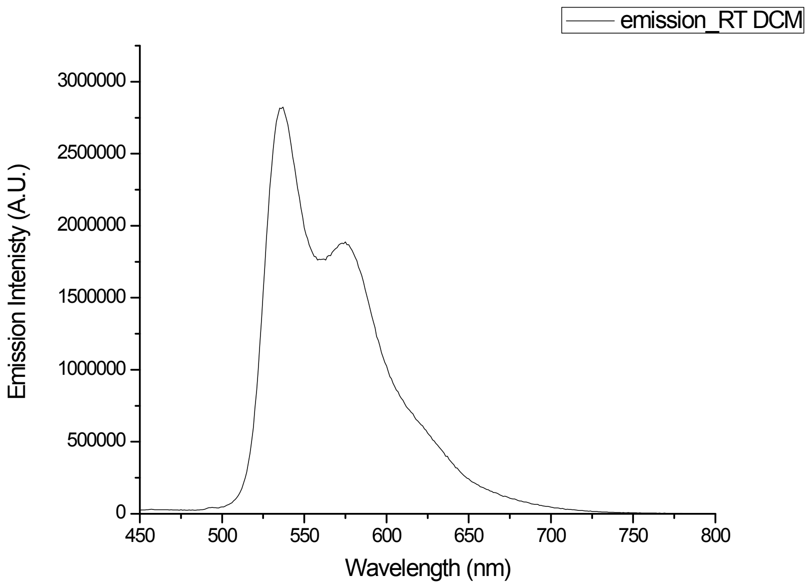

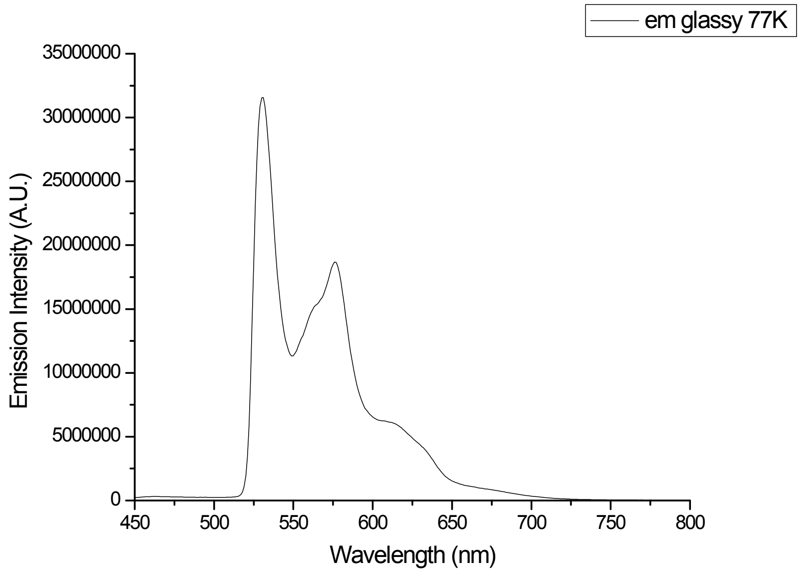

Figure S3. Emission spectra of **3-endo** in dichloromethane (DCM) at room temperature (top) and in a glassy medium at 77K (bottom); λ_{exc} = 430 nm.

Computational Details.

In this work, the hybrid density functional, PBE0,[3] was employed for all calculations using the program package G09.[4] The 6-31G* basis set[5] is used for all atoms except Au, which is described by the Stuttgart relativistic pseudopotential and its accompanying basis set (ECP60MWB).[6] Solvent effect was also included by means of the polarizable continuum model (PCM).[7] Geometry optimizations of the singlet ground state (S_0) and the lowest triplet excited state (T_1) were respectively carried out using restricted and unrestricted density functional theory (i.e. RDFT and UDFT) formalism without symmetry constraints. Frequency calculations were performed on the optimized structures to ensure that they are minimum energy structures by the absence of imaginary frequency (i.e. NImag = 0). Stability calculations were also performed for all the optimized structures to ensure that all the wavefunctions obtained are stable.

(a) ***SS-PCM energy calculations.*** Vertical transition energies were computed using the linear response approximation for optical absorption calculations, but the state specific approach for emissions. [8] In an absorption process, the solvent is in equilibrium solvation with the ground state electron density but non-equilibrium solvation with the excited state electron density. Thus, LR-TDDFT should give reasonable estimate of the absorption energies. However, as mentioned in the main text, SS-TDDFT is more adequate for calculations involving an emission process.

Within the state-specific (SS) approach, the equilibrium solvation of the T_1 excited state at its equilibrium geometry is written out via "NonEq=write":

```
%oldchk=cncauccphome_pbe0_t1.chk          chk file of optimized T1 that is confirmed to be stable
%chk=cncauccphome_pbe0_t1ss.chk
#p pbe1pbe/chkbas geom=check guess=read scrf=(solvent=dichloromethane,read)
nosymm pop=full

Save solvent reaction field in equilibrium with T1 density
at its optimized geometry to the checkpoint file, cncauccphome_pbe0_t1ss.chk

0 3

NonEq=write
```

The ground state and the singlet excited state energy could then be computed with non-equilibrium solvation using "NonEq = read"; for example, for the singlet excited state energy calculation,

```
%oldchk=cncauccphome_pbe0 _t1ss.chk
%chk=cncauccphome_pbe0_td-s1ss.chk
#p rpbe1pbe/chkbas geom=check guess=read td=(singlets,nstates=3,root=1)
scrf=(solvent=dichloromethane,read,externaliteration) nosymm pop=full

S1 energy and density with non-eqm solvation, i.e. fast polarization is solved self-consistently in a
state-specific way for S1 and the slow polarization is frozen at that in eqm with T1 density
```

```
0 1

NonEq=read
```

The energy that should be extracted would appear near the end of the output file:

```
-----------------------------------------------------------------
 After PCM corrections, the energy is  -1265.80793189     a.u.
 -----------------------------------------------------------------
```

That is, this is the S_1 energy at the T_1 optimized geometry, with non-equilibrium solvation done in an SS approach.

Similarly, for the ground state energy calculation with non-equilibrium solvation at the T_1 excited state geometry,

```
%oldchk=cncauccphome_pbe0 _t1ss.chk
%chk=cncauccphome_pbe0_s0ss.chk
#p rpbe1pbe/chkbas geom=check guess=read
scrf=(solvent=dichloromethane,read) nosymm pop=full

S0 state non-eqm solvation at optimized T1 excited state geometry

0 1

NonEq=read
```

And the energy of the ground state from a non-equilibrium solvation in solution is:

SCF Done: E(RPBE1PBE) = **-1265.92332980**   A.U. after   13 cycles

(b) ***Radiative decay rate calculations.*** The spin wavefunctions of the triplet sub-states T_1^{α} are expressed along the three Cartesian coordinates, x, y, and z as:

$$\sigma_T{}^x = \frac{1}{\sqrt{2}}(\beta\beta - \alpha\alpha)$$

$$\sigma_T{}^y = \frac{i}{\sqrt{2}}(\beta\beta + \alpha\alpha)$$

$$\sigma_T{}^z = \frac{1}{\sqrt{2}}(\alpha\beta + \beta\alpha)$$

and the singlet spin wavefunction as:

$$\sigma_S = \frac{1}{\sqrt{2}}(\alpha\beta - \beta\alpha)$$

Phosphorescence, being a spin-forbidden process, borrows its emission intensity by first-order perturbative interactions with the singlet excited states via spin-orbit coupling (SOC). Therefore, the singlet excited state energies should also be evaluated at *non-equilibrium solvation* with the emitting triplet excited state electronic density. Therefore, in principle, the SS approach is more appropriate than the LR approach for calculating the energy difference between the singlet and triplet excited states in eq.(9) of the main text. If the singlet excited state energies are computed within the LR-TDDFT, the singlet excited state energies are obtained either through *non-equilibrium solvation with ground state electronic density* or *equilibrium solvation with the singlet excited state of interest* (by specifying the "root" in the LR-TDDFT calculation). In either case, the energies obtained are not the solvent response to the *emitting triplet excited state* electronic density.

To calculate the radiative decay rate constant using the SS-TDDFT results, the metal coefficients (c_{jk}), the CI coefficients (a_j), and the transition dipole moments (M_x, M_y, M_z) of each singlet excited state (S_m) of interest, could be extracted from the output files of the SS-TDDFT calculations at the T_1 optimized geometry at the last step of the iterative procedure for each S_m excited state considered. For the singlet-triplet energy gap, $(E(S_m) - E(T_1))$, it should be emphasized that this is not the energy difference for the transitions , S_m → S_0 and T_1 → S_0, from TDDFT calculations (i.e., the section where the CI coefficients are extracted), but those with PCM corrections added, i.e., the energies mentioned in the previous section ("After PCM corrections…").

In addition, the singlet excited state energies may shift, depending on the electron density of the emitting T_1 state. This is particularly important if there is a large difference in dipole moments between the emitting T_1 and S_m excited states in a polar medium. This means that one has to do a SS-TDDFT calculation for *each* singlet excited state S_m. For example, if one wants to include the first ten singlet excited states in estimating the radiative decay rate constant through eq. (9), then one has to do a SS-TDDFT calculation for each singlet excited state, i.e. a total of ten SS-TDDFT jobs (with root = 1, 2, …, 10 in the "NonEq=read" step). This could be quite a lengthy task; hence, for simplicity, only the closest-lying singlet excited state(s) that could have effective SOC with the T_1 excited state were included in estimating k_r as these states would dominate in the calculation of radiative decay rate constant (further details for the specific complexes would be given in a later section).

The SOC matrix elements are listed in Table S4.

Table S4. SOC matrix elements between the $^3(d_{ki}\pi^*)$ and $^1(d_{lj}\pi^*)$ states; $\langle S_n|H_{SOC}|T_1\rangle$

$\langle S_n\|H_{SOC}\|T_1\rangle$	$^3(d_{z^2}\pi^*)$	$^3(d_{x^2-y^2}\pi^*)$	$^3(d_{xy}\pi^*)$	$^3(d_{xz}\pi^*)$	$^3(d_{yz}\pi^*)$
$^1(d_{z^2}\pi^*)$	0	0	0	$\frac{i\sqrt{3}}{2}\zeta c_{xz}c_{z^2}$	$-\frac{i\sqrt{3}}{2}\zeta c_{yz}c_{z^2}$
$^1(d_{x^2-y^2}\pi^*)$	0	0	$i\zeta c_{x^2-y^2}c_{xy}$	$-\frac{i}{2}\zeta c_{x^2-y^2}c_{xz}$	$-\frac{i}{2}\zeta c_{x^2-y^2}c_{yz}$
$^1(d_{xy}\pi^*)$	0	$-i\zeta c_{x^2-y^2}c_{xy}$	0	$\frac{i}{2}\zeta c_{xy}c_{xz}$	$-\frac{i}{2}\zeta c_{xy}c_{yz}$
$^1(d_{xz}\pi^*)$	$-\frac{i\sqrt{3}}{2}\zeta c_{xz}c_{z^2}$	$\frac{i}{2}\zeta c_{x^2-y^2}c_{xz}$	$-\frac{i}{2}\zeta c_{xy}c_{xz}$	0	$\frac{i}{2}\zeta c_{xz}c_{yz}$

$^1(d_{yz}\pi^*)$	$\frac{i\sqrt{3}}{2}\zeta c_{yz}c_{z^2}$	$\frac{i}{2}\zeta c_{x^2-y^2}c_{yz}$	$\frac{i}{2}\zeta c_{xy}c_{yz}$	$-\frac{i}{2}\zeta c_{xz}c_{yz}$	0

x-component: magenta

y-component: brown

z-component: green

(c) ***Non-radiative decay rates.*** To calculate the Huang-Rhys factor, S_j, and to simulate the emission spectrum for $\langle \tilde{\nu} \rangle_{fcf}$, a Franck-Condon calculation is done using the keyword "freq=fc". One could request the printing of the "shift vector" (which relates to $\sqrt{m_j}\Delta Q_j$) for the computation of S_j. For a more detailed theoretical background for the Franck-Condon calculation implemented in the G09 program, please consult the references cited in G09 and its document titled "Vibrationally-resolved electronic spectra in Gaussian 09".

Following our previous work,[9] we have grouped the normal modes into 5 sets:

$\omega_{lf1} \le 200$ cm^{-1}, $200 < \omega_{lf2} \le 1000$ cm^{-1}, $1000 < \omega_m \le 1800$ cm^{-1}, $\omega_{C\equiv C}$, and $\omega_{hf} \ge 3000$ cm^{-1}

For the non-radiative decay rate calculations, k_BT is assumed to be ~200 cm^{-1}.

For ^{3}IL → S_0 transition, the single-mode expression (eq.(11) of the main text) is used; however, for ^{3}LLCT → S_0 transition, as both aromatic CC/CN stretching and C≡C stretching normal modes could act as effective accepting modes, the two-mode expression is used instead:[10]

$$k_{nr}(T_1 \rightarrow S_0) = \frac{\frac{2\pi \langle T_1 | H_{SOC} | S_0 \rangle^2}{\hbar}}{(4\pi\lambda_s k_BT)^{\frac{1}{2}}} \exp\left(-S_C - S_M \coth\left(\frac{\hbar\omega_M}{2k_BT}\right)\right) \times \sum_{n_C}^{\infty}\sum_{n_M}^{\infty} exp\left(-\frac{(\Delta E - n_M\hbar\omega_M - n_C\hbar\omega_C - \lambda_s)^2}{4\lambda_s k_BT}\right) \times \frac{S_C^{\,n_C}}{n_C!}\exp\left(\frac{n_M\hbar\omega_M}{2k_BT}\right) I_{n_M}\left\{S_M \operatorname{csch}\left(\frac{\hbar\omega_M}{2k_BT}\right)\right\}$$

Here, S_C and n_C are respectively the Huang-Rhys factor and change in the vibrational quantum number of the C≡C stretching normal mode ($\hbar\omega_C = \hbar\omega_{C\equiv C}$ ~ 2200–2300 cm^{-1}) and I_{nM} is the modified Bessel function of order n_M.

[1].W.-P. To, K. T. Chan, G. S. M. Tong, C. Ma, W.-M. Kwok, X. Guan, K.-H. Low and C.-M. Che, *Angew. Chem. Int. Ed.*, 2013, **52**, 6648-6652.
[2].in *CCDC 1034529 (**3-endo**) contains the supplementary crystallographic data for this manuscript. The data can be obtained from The Cambridge Crystallographic Data Centre free of charge. URL: http://www.ccdc.cam.ac.uk/data_request/cif*

[3].C. Adamo and V. Barone, *J. Chem. Phys.*, 1999, **110**, 6158-6169.
[4].M. J. Frisch, G. W. Trucks, H. B. Schlegel, G. E. Scuseria, M. A. Robb, J. R. Cheeseman, G. Scalmani, V. Barone, B. Mennucci, G. A. Petersson, H. Nakatsuji, M. Caricato, X. Li, H. P. Hratchian, A. F. Izmaylov, J. Bloino, G. Zheng, J. L. Sonnenberg, M. Hada, M. Ehara, K. Toyota, R. Fukuda, J. Hasegawa, M. Ishida, T. Nakajima, Y. Honda, O. Kitao, H. Nakai, T. Vreven, J. Montgomery, J. A.; ,

J. E. Peralta, F. Ogliaro, M. Bearpark, J. J. Heyd, E. Brothers, K. N. Kudin, V. N. Staroverov, R. Kobayashi, J. Normand, K. Raghavachari, A. Rendell, J. C. Burant, S. S. Iyengar, J. Tomasi, M. Cossi, N. Rega, J. M. Millam, M. Klene, J. E. Knox, J. B. Cross, V. Bakken, C. Adamo, J. Jaramillo, R. Gomperts, R. E. Stratmann, O. Yazyev, A. J. Austin, R. Cammi, C. Pomelli, J. W. Ochterski, R. L. Martin, K. Morokuma, V. G. Zakrzewski, G. A. Voth, P. Salvador, J. J. Dannenberg, S. Dapprich, A. D. Daniels, Ö. Farkas, J. B. Foresman, J. V. Ortiz, J. Cioslowski and D. J. Fox, *Gaussian 09 (Revision C.01),* Gaussian, Inc., Wallingford CT, 2009.

[5].(a)M. M. Francl, W. J. Petro, W. J. Hehre, J. S. Binkley, M. S. Gordon, D. J. DeFree and J. A. Pople, *J. Chem. Phys.*, 1982, **77**, 3654; (b)P. C. Hariharan and J. A. Pople, 1973, **28**, 123.

[6].(a)D. Andrae, U. Haeussermann, M. Dolg, H. Stoll and H. Preuss, *Theor. Chim. Acta*, 1990, **77**, 123-141; (b)J. M. L. Martin and A. Sundermann, *J. Chem. Phys.*, 2001, **114**, 3408-3420.

[7].M. Cossi, G. Scalmani, N. Rega and V. Barone, *J. Chem. Phys.*, 2002, **117**, 43-54.

[8].R. Improta, V. Barone, G. Scalmani and M. J. Frisch, *J. Chem. Phys.*, 2006, **125**, 054103: 054101-054109.

[9].G. S. M. Tong, P. K. Chow, W.-P. To, W.-M. Kwok and C.-M. Che, *Chem. Eur. J.*, 2014, **20**, 6433-6443.

[10].B. S. Brunschwig and N. Sutin, *Comments Inorg. Chem.*, 1987, **6**, 209-235.

[11].A. Dreuw and M. Head-Gordon, *Chem. Rev.*, 2005, **105**, 4009-4037.

[12].D. Jacquemin, E. A. Perpete, I. Ciofini and C. Adamo, *Acc. Chem. Res.*, 2009, **42**, 326-334.

[13].T. Yanai, a. P. Tew and N. C. Handy, *Chem. Phys. Lett.*, 2004, **393**, 51-57.

Table S5. Calculated low-lying singlet and triplet excited state energies (λ / nm), the associated oscillator strengths (*f*) and the nature of the transitions at the optimized ground state (S_0) geometries of complex **1** in dichloromethane (DCM) by LR-PCM TD-PBE0 method. The values in the parentheses are the % contributions of that particular configuration state function (CSF).

HOMO is 104					
	E (cm-1)	λ (nm)	*f*	Major contribs	Minor contribs
T1	22041	454	0.0000	H-4->LUMO (13%), H-1->L+1 (46%), HOMO->LUMO (17%)	H-10->L+10 (2%), H-9->LUMO (2%), H-3->L+1 (4%), HOMO->L+2 (3%), HOMO->L+4 (2%)
T2	22108	452	0.0000	H-1->LUMO (83%)	H-6->L+1 (2%), H-4->L+1 (4%), H-4->L+7 (2%), H-3->L+4 (2%)
T3	22670	441	0.0000	H-1->L+1 (15%), HOMO->LUMO (44%), HOMO->L+2 (20%)	H-5->L+5 (2%), H-4->LUMO (5%), HOMO->L+4 (6%)
**S1**	**25484**	**392**	**0.2510**	**HOMO->LUMO (98%)**	
T4	26853	372	0.0000	H-2->LUMO (83%)	H-7->LUMO (3%), HOMO->LUMO (4%), HOMO->L+2 (4%), HOMO->L+4 (2%)
T5	27190	368	0.0000	H-2->LUMO (11%), HOMO->LUMO (31%), HOMO->L+2 (27%), HOMO->L+4 (13%)	H-5->L+5 (7%), HOMO->L+6 (3%)
**S2**	**27220**	**367**	**0.0519**	**H-1->LUMO (97%)**	
**S3**	**28076**	**356**	**0.0029**	**H-2->LUMO (97%)**	

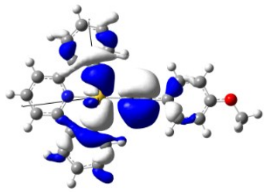
MO102 (H-2) -6.6eV 11%

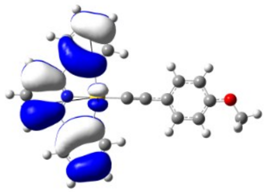
MO103 (H-1) -6.47eV 4%

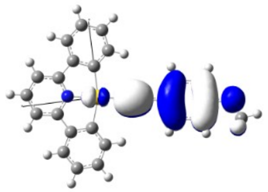
MO104 (HOMO) -5.84eV 4%

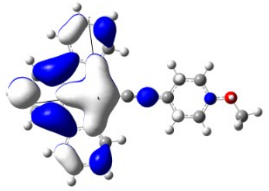
MO105 (LUMO) -2.12eV 9%

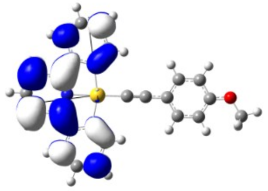
MO106 (L+1) -1.55eV

Figure S4. Frontier MOs of complex **1** at the optimized S_0 geometry from the LR-PCM TD-PBE0 calculation. The orbital energy and the Au character (in %) is also displayed.

Table S6. Calculated low-lying singlet and triplet excited state energies (λ / nm) , the associated oscillator strengths (*f*) and the nature of the transitions at the optimized ground state (S_0) geometries of complex **2** in dichloromethane (DCM) by LR-PCM TD-PBE0 method. The values in the parentheses are the % contributions of that particular configuration state function (CSF).

HOMO is 169					
No.	E (cm-1)	λ (nm)	f	Major contribs	Minor contribs
T1	19149	522	0	H-1->LUMO (11%), H-1->L+1 (47%), H-1->L+2 (12%)	H-6->LUMO (2%), H-2->LUMO (3%), H-2->L+1 (3%), H-2->L+2 (3%), H-1->L+3 (2%), HOMO->LUMO (2%), HOMO->L+1 (3%)
T2	22044	454	0	H-2->LUMO (24%), H-2->L+1 (19%)	H-7->L+8 (2%), H-6->LUMO (5%), H-6->L+1 (4%), H-5->LUMO (6%), H-1->LUMO (9%), H-1->L+2 (2%), HOMO->LUMO (9%), HOMO->L+5 (2%)
T3	22522	444	0	HOMO->LUMO (49%), HOMO->L+5 (11%)	H-8->L+7 (3%), H-6->LUMO (2%), H-2->LUMO (6%), H-2->L+1 (3%), H-1->L+1 (2%), HOMO->L+2 (5%), HOMO->L+3 (8%)
T4	24715	405	0	H-5->LUMO (15%), H-2->LUMO (24%), H-2->L+1 (15%), H-1->L+2 (10%)	H-12->LUMO (2%), H-5->L+3 (3%), H-4->L+6 (3%), H-1->LUMO (6%), H-1->L+1 (4%), HOMO->L+3 (3%), HOMO->L+5 (4%)
**S1**	**24954**	**401**	**0.2737**	**HOMO->LUMO (97%)**	
T5	25090	399	0	H-2->LUMO (15%), H-1->LUMO (55%)	H-5->LUMO (4%), H-5->L+3 (2%), H-4->L+6 (2%), H-2->L+2 (2%), H-1->L+2 (6%)
T6	26062	384	0	H-5->LUMO (18%), H-2->LUMO (11%), H-2->L+1 (21%)	H-8->L+7 (2%), H-6->LUMO (3%), H-5->L+3 (5%), H-4->L+6 (8%), HOMO->LUMO (7%), HOMO->L+2 (2%), HOMO->L+3 (3%), HOMO->L+5 (6%)
T7	27003	370	0	H-3->LUMO (10%), HOMO->LUMO (25%), HOMO->L+5 (16%)	H-8->L+7 (5%), H-5->LUMO (8%), H-5->L+3 (2%), H-4->L+6 (4%), H-1->LUMO (6%), HOMO->L+2 (4%), HOMO->L+3 (6%), HOMO->L+8 (2%)
**S2**	**27050**	**370**	**0.2623**	**H-2->LUMO (27%), H-1->LUMO (68%)**	
T8	27257	367	0	H-3->LUMO (81%)	H-9->LUMO (2%), H-5->LUMO (2%), H-3->L+1 (2%), H-3->L+3 (2%), HOMO->LUMO (3%), HOMO->L+5 (2%)
**S3**	**27609**	**362**	**0.028**	**H-2->LUMO (68%), H-1->LUMO (26%)**	**HOMO->L+1 (2%)**
**S4**	**28139**	**355**	**0.0021**	**H-3->LUMO (96%)**	

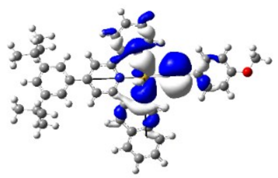

MO166 (H-3) -6.59eV 11%

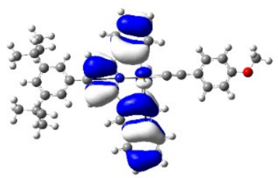

MO167 (H-2) -6.5eV 5%

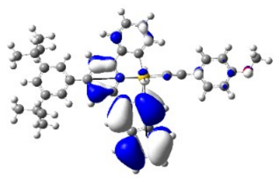

MO168 (H-1) -6.23eV 1%

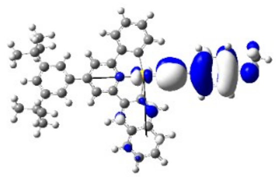

MO169 (HOMO) -5.81eV 4%

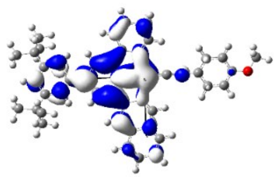

MO170 (LUMO) -2.19eV 7%

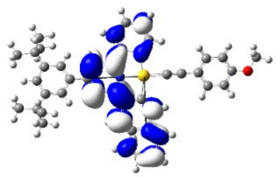

MO171 (L+1) -1.74eV 1%

Figure S5. Frontier MOs of complex **2** at the optimized S_0 geometry from the LR-PCM TD-PBE0 calculation. The orbital energy and the Au character (in %) is also displayed.

Table S7. Calculated low-lying singlet and triplet excited state energies (λ / nm) with λ > 300 nm, the associated oscillator strengths (*f*) and the nature of the transitions at the optimized ground state (S_0) geometries of complex **3-exo** in dichloromethane (DCM) by LR-PCM TD-PBE0 method. The values in the parentheses are the % contributions of that particular configuration state function (CSF).

HOMO is 227					
	E (cm-1)	λ (nm)	f	Major contribs	Minor contribs
T1	19235	520	0	H-1->LUMO (55%), H-1->L+1 (27%)	
T2	21713	461	0	H-3->LUMO (14%), H-1->LUMO (18%), H-1->L+1 (20%)	H-5->LUMO (7%), H-5->L+1 (7%), H-3->L+1 (9%), HOMO->LUMO (8%)
T3	22427	446	0	HOMO->LUMO (54%)	H-9->L+8 (2%), H-3->LUMO (2%), H-1->L+1 (5%), HOMO->L+2 (9%), HOMO->L+3 (8%), HOMO->L+5 (7%), HOMO->L+8 (2%)
**S1**	**24447**	**409**	**0.1645**	**H-1->LUMO (84%), HOMO->LUMO (11%)**	**H-3->LUMO (3%)**
**S2**	**24932**	**401**	**0.3078**	**H-1->LUMO (10%), HOMO->LUMO (87%)**	
T4	25514	392	0	H-6->LUMO (34%), H-4->L+6 (10%)	H-6->L+2 (9%), H-5->LUMO (2%), H-4->L+4 (2%), H-3->LUMO (3%), H-1->L+1 (4%), H-1->L+3 (2%), HOMO->L+2 (4%), HOMO->L+3 (2%), HOMO->L+5 (3%)
T5	26681	375	0	H-2->LUMO (92%)	H-10->LUMO (2%), H-2->L+2 (2%)
T6	26759	374	0	HOMO->LUMO (20%), HOMO->L+5 (10%)	H-12->LUMO (2%), H-9->L+7 (2%), H-9->L+8 (2%), H-8->LUMO (2%), H-3->L+1 (9%), H-1->LUMO (5%), H-1->L+1 (2%), H-1->L+2 (3%), H-1->L+3 (4%), HOMO->L+1 (2%), HOMO->L+2 (6%), HOMO->L+3 (8%), HOMO->L+7 (3%), HOMO->L+8 (2%)
T7	26981	371	0	HOMO->LUMO (11%)	H-12->LUMO (2%), H-11->LUMO (2%), H-9->L+8 (2%), H-6->LUMO (8%), H-6->L+1 (2%), H-6->L+2 (4%), H-5->L+1 (3%), H-4->L+6 (7%), H-3->LUMO (2%), H-3->L+1 (6%), H-1->LUMO (6%), H-1->L+1 (4%), H-1->L+2 (3%), H-1->L+3 (4%), HOMO->L+2 (3%), HOMO->L+3 (3%), HOMO->L+5 (4%)
T8	27314	366	0	H-3->LUMO (40%), H-1->L+1 (18%)	H-12->L+1 (4%), H-5->LUMO (8%), H-3->L+1 (2%), H-1->LUMO (5%), H-1->L+3 (2%), H-1->L+10 (2%)
**S3**	**27705**	**361**	**0.0001**	**H-2->LUMO (97%)**	

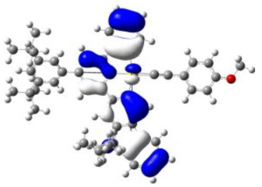 MO224 (H-3) -6.75eV 5%	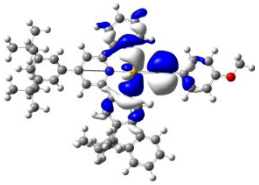 MO225 (H-2) -6.57eV 11%	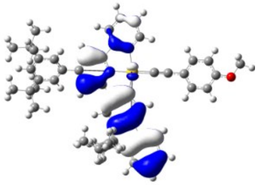 MO226 (H-1) -6.02eV 1%
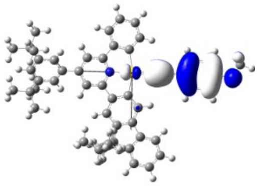 MO227 (HOMO) -5.82eV 4%	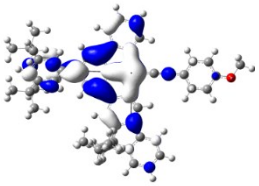 MO228 (LUMO) -2.2eV 7%	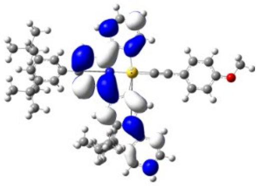 MO229 (L+1) -1.63eV 1%

Figure S6. Frontier MOs of complex **3-exo** at the optimized S_0 geometry from the LR-PCM TD-PBE0 calculation. The orbital energy and the Au character (in %) is also displayed.

Table S8. Calculated low-lying singlet and triplet excited state energies (λ / nm) with λ > 300 nm, the associated oscillator strengths (*f*) and the nature of the transitions at the optimized ground state (S_0) geometries of complex **3-endo** in dichloromethane (DCM) by LR-PCM TD-PBE0 method. The values in the parentheses are the % contributions of that particular configuration state function (CSF).

HOMO is 227					
	E (cm-1)	λ (nm)	f	Major contribs	Minor contribs
T1	19337	517	0	H-1->LUMO (54%), H-1->L+1 (28%)	H-12->LUMO (2%), H-1->L+2 (2%), H-1->L+4 (2%)
T2	21609	463	0	H-2->LUMO (11%), H-1->LUMO (18%), H-1->L+1 (22%)	H-7->L+1 (3%), H-6->LUMO (4%), H-4->LUMO (5%), H-4->L+1 (4%), H-3->LUMO (3%), H-3->L+1 (3%), H-2->L+1 (7%), HOMO->LUMO (5%)
T3	22314	448	0	HOMO->LUMO (69%)	H-9->L+8 (2%), H-2->LUMO (2%), H-1->L+1 (2%), HOMO->L+2 (4%), HOMO->L+3 (3%), HOMO->L+4 (4%), HOMO->L+5 (4%), HOMO->L+6 (2%)
**S1**	**23499**	**426**	**0.0671**	**HOMO->LUMO (96%)**	
**S2**	**24564**	**407**	**0.2505**	**H-1->LUMO (93%)**	**H-2->LUMO (3%)**
T4	25235	396	0	HOMO->LUMO (17%), HOMO->L+3 (11%), HOMO->L+5 (16%)	H-9->L+8 (7%), H-6->LUMO (3%), HOMO->L+2 (6%), HOMO->L+4 (9%), HOMO->L+6 (8%), HOMO->L+7 (8%), HOMO->L+8 (2%)
T5	25640	390	0	H-6->LUMO (27%), H-6->L+2 (10%), H-3->LUMO (15%)	H-7->LUMO (4%), H-5->L+5 (5%), H-5->L+6 (8%), H-4->LUMO (3%), H-2->LUMO (2%), H-1->L+1 (2%), HOMO->LUMO (2%)
T6	26691	375	0	H-2->LUMO (35%), H-1->LUMO (17%), H-1->L+1 (17%)	H-12->L+1 (2%), H-3->LUMO (6%), H-2->L+2 (2%)
T7	26978	371	0	H-2->LUMO (11%), H-2->L+1 (15%)	H-12->LUMO (4%), H-10->LUMO (2%), H-7->L+1 (3%), H-6->LUMO (4%), H-6->L+2 (2%), H-5->L+5 (2%), H-5->L+6 (3%), H-4->L+9 (2%), H-3->L+1 (5%), H-1->LUMO (3%), H-1->L+2 (7%), H-1->L+4 (6%), H-1->L+9 (4%)
T8	28155	355	0	H-7->LUMO (20%), H-4->LUMO (20%), HOMO->L+1 (11%)	H-7->L+1 (2%), H-6->LUMO (4%), H-4->L+1 (3%), H-3->LUMO (7%), H-2->LUMO (7%), H-1->L+1 (5%), H-1->L+2 (3%)
T9	28425	352	0	H-3->LUMO (16%), HOMO->L+1 (64%)	H-6->LUMO (3%), H-2->LUMO (6%)
**S3**	**28623**	**349**	**0.0053**	**HOMO->L+1 (97%)**	
T10	28716	348	0	H-4->LUMO (13%), H-3->LUMO (33%), HOMO->L+1 (19%)	H-7->LUMO (9%), H-2->LUMO (5%), H-1->L+1 (2%), HOMO->LUMO (2%)
**S4**	**29756**	**336**	**0.0356**	**H-2->LUMO (87%)**	**H-3->LUMO (2%), H-1->LUMO (2%)**

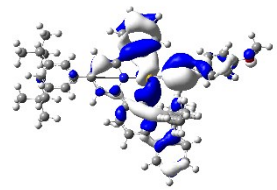 MO224 (H-3) -6.74eV 8%	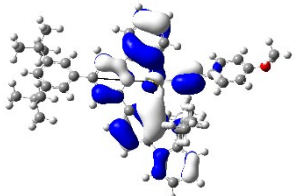 MO225 (H-2) -6.71eV 7%	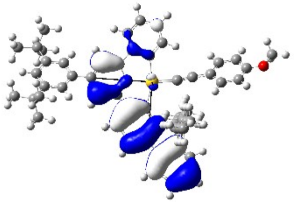 MO226 (H-1) -5.98eV 1%
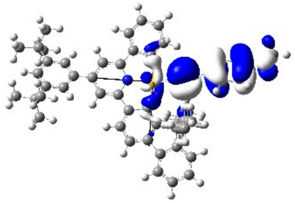 MO227 (HOMO) -5.72eV 4%	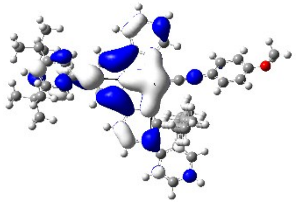 MO228 (LUMO) -2.19eV 8%	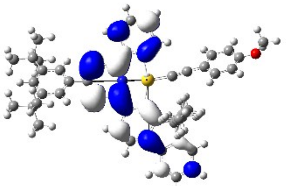 MO229 (L+1) -1.6eV %

Figure S7. Frontier MOs of complex **3-endo** at the optimized S_0 geometry from the LR-PCM TD-PBE0 calculation. The orbital energy and the Au character (in %) is also displayed.

Table S9. CI coefficients of the HOMO → LUMO and H–1 → LUMO transitions from the LR-PCM TD-PBE0 calculation of the first singlet excited state at the optimized ground state geometries of complexes depicted in Chart 1 of the main text.

CI coefficients of S_1	**1**	**2**	**3-exo**	**3-endo**
HOMO → LUMO	0.70139	0.69767	0.23644	0.68544
H–1 → LUMO	0	0.07379	0.64616	0.09113

Radiative decay rate calculation results. For the ^{3}IL excited state, the singlet excited state that could have appreciable SOC is ^{1}LLCT as the HOMO and H−1 are orthogonal. For complexes **1** and **2**, the lowest-lying ^{1}LLCT excited state is S_1 and k_r is calculated for m = 1 for these two complexes using eq.(9) of the main text. On the other hand, for complexes **3-exo** and **3-endo**, the closest-lying ^{1}LLCT excited state is S_2; hence, for these two complexes, both S_1 and S_2 are included in the k_r calculations.

Similarly, for the ^{3}LLCT excited state of complexes **1** and **3-endo**, the singlet excited state that could have appreciable SOC is ^{1}IL/^{1}MLCT. For complex **1**, S_3 is of the character ^{1}IL/^{1}MLCT from the NonEq SS-TDDFT calculation, and it is found to be mainly of "H−4" $\rightarrow$ L transition ("H−4" in the NEQ SS-TDDFT calculation of the S_1 excited state; this orbital becomes H−1 in the NonEq SS-TDDFT calculation of the S_3 excited state). But S_4, though also a ^{1}LLCT and is derived from a "H−2" $\rightarrow$ L transition ("H−2" in the NEQ SS-TDDFT calculation of the S_1 excited state; this orbital becomes H−1 in the NonEq SS-TDDFT calculation of the S_4 excited state), could also has significant SOC with the ^{3}LLCT excited state of complex **1** because this H-2 has the Au(d) orbital orthogonal to the HOMO, see Figure S8. Therefore, in estimating the k_r of ^{3}LLCT $\rightarrow$ S_0 of complex **1** using eq.(9) of the main text, both m = 3 and 4 are considered to be the main states that contribute to the radiative decay rate.

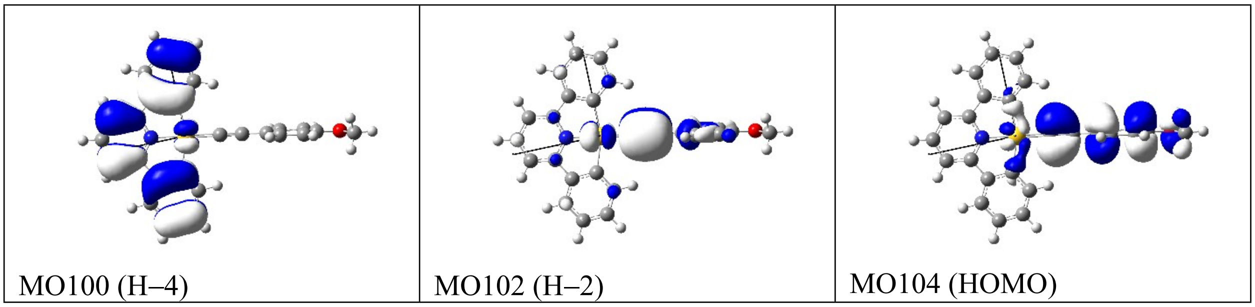

Figure S8. MO surfaces relevant to the SOC calculation of the ^{3}LLCT excited state of complex **1**. The relative MO orders are those obtained from a NEQ SS-TDDFT calculation of the S_1 excited state.

Likewise, for the ^{3}LLCT excited state of complex **3-endo**, both the ^{1}IL/^{1}MLCT and ^{1}LLCT singlet excited states were considered to be the two major S_m excited states contributing to the radiative decay rates. Therefore, NonEq SS-TDDFT calculations were also performed for the S_3 (mainly of ^{1}IL/^{1}MLCT character and is derived from H−1 $\rightarrow$ L and H−5 $\rightarrow$ L transitions) and S_4 (mainly of ^{1}LLCT character and is derived from "H−3" $\rightarrow$ L transition; "H−3" in the NEQ SS-TDDFT calculation of the S_1 excited state and becomes H−2 in the NonEq SS-TDDFT calculation of the S_4 excited state)

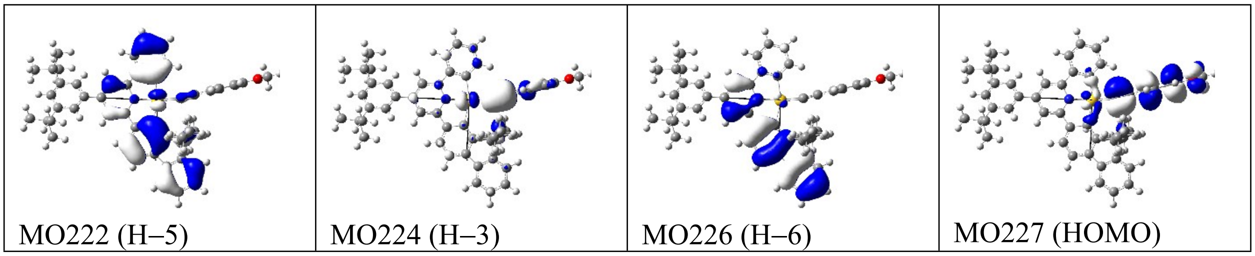

Figure S9. MO surfaces relevant to the SOC calculation of the ^{3}LLCT excited state of complex **3-endo**. The relative MO orders are those obtained from a NEQ SS-TDDFT calculation of the S_1 excited state.

Non-radiative decay rate calculations. Basically, the FCF's were calculated using eq.(11) of the main text. For complex **3-endo**, as the Huang-Rhys factor for the low-frequency modes, i.e., $\sum_{j \in lf1} S_j$ ($\omega_{lf1} \le 200$ cm^{-1}) > 1 (*vide infra*), eq.(S1) would be used:

$$k_{nr}(T_1 \to S_0) = \frac{2\pi \langle T_1 | H_{SOC} | S_0 \rangle^2}{\hbar \, (4\pi \lambda k_B T)^{1/2}} \sum_{n_M}^{\infty} \exp\left(- \frac{(\Delta E - n_M \hbar\omega_M - \lambda_s)^2}{4\lambda k_B T} \right) \exp(-S_M) \frac{S_M^{\,n_M}}{n_M!} \tag{S1}$$

where λ includes contributions from both solvent modes and the low-frequency normal modes ($\omega_{lf1} \le 200$ cm^{-1}), i.e.,

$$\lambda = \lambda_s + \lambda_{lf1} \tag{S2a}$$

$$\lambda_{lf1} = \sum_{j \in lf1} S_j \hbar\omega_j \tag{S2b}$$

Table S10. Average normal mode ω_A (cm^{-1}) and the corresponding Huang-Rhys factor S_A and reorganization energy λ_A (cm^{-1}) of ^{3}IL → S_0 transition of complex **1**.

ω_j	ω_A	S_A	λ_A
$\omega_{lf1} \le 200$ cm^{-1}	76	0.21	15.9
$200 < \omega_{lf2} \le 1000$ cm^{-1}	579	0.56	327
$1000 < \omega_M \le 1800$ cm^{-1}	1467	1.75	2570
$\omega_{hf} \ge 3000$ cm^{-1}	3182	0.00	6.43

Table S11. Average normal mode ω_A (cm^{-1}) and the corresponding Huang-Rhys factor S_A and reorganization energy λ_A (cm^{-1}) of ^{3}IL → S_0 transition of complex **2**.

ω_j	ω_A	S_A	λ_A
$\omega_{lf1} \le 200$ cm^{-1}	18	0.90	16.2
$200 < \omega_{lf2} \le 1000$ cm^{-1}	373	0.99	368
$1000 < \omega_M \le 1800$ cm^{-1}	1508	1.47	2223
$\omega_{hf} \ge 3000$ cm^{-1}	3114	0.00	10.3

Table S12. Average normal mode ω_A (cm^{-1}) and the corresponding Huang-Rhys factor S_A and reorganization energy λ_A (cm^{-1}) of ^{3}IL → S_0 transition of complex **3-exo**.

ω_j	ω_A	S_A	λ_A
$\omega_{lf1} \le 200$ cm^{-1}	72	0.76	54.4
$200 < \omega_{lf2} \le 1000$ cm^{-1}	619	0.70	435
$1000 < \omega_M \le 1800$ cm^{-1}	1483	1.29	1917
$\omega_{hf} \ge 3000$ cm^{-1}	3147	0.00	2.13

Table S13. Average normal mode ω_A (cm^{-1}) and the corresponding Huang-Rhys factor S_A and reorganization energy λ_A (cm^{-1}) of ^{3}IL → S_0 transition of complex **3-endo**.

ω_j	ω_A	S_A	λ_A
$\omega_{lf1} \leq 200$ cm^{-1}	53	1.33	69.9
$200 < \omega_{lf2} \leq 1000$ cm^{-1}	625	0.74	462
$1000 < \omega_M \leq 1800$ cm^{-1}	1501	1.27	1906
$\omega_{hf} \geq 3000$ cm^{-1}	3137	0.00	1.16

Is PBE0 calculation reliable in predicting the relative energies of the ^{3}LLCT and ^{3}IL excited states?

Global hybrid functionals are known to fail in zero-overlap charge transfer transition.[11] However, it has also been shown that TD-PBE0 can also provide accurate descriptions with partial charge transfer character, without resorting to range-separated hybrid density functionals.[12] To confirm that our present calculation results using the PBE0 functional are valid, we have also done SS-TDDFT calculation using the same basis set but a long-range corrected density functional, CAM-B3LYP[13] for complex 1. It was found that with the CAM-B3LYP functional at the PBE0 optimized triplet excited state geometries, the ^{3}LLCT excited state is only ~0.04 eV above the ^{3}IL excited state using the unrestricted formalism (UDFT); when SS-TDDFT was employed, the ^{3}LLCT excited state was found to be ~0.05 eV below the ^{3}IL excited state. That is, even with a long-range corrected functional, the TD-CAMB3LYP results also showed that the ^{3}LLCT excited state is slightly lower-lying than the ^{3}IL excited state. Therefore, the ^{3}LLCT excited state is a thermally accessible excited state that contributes to the fast non-radiative decay rate of complex **1**.

Table S14. Optimized S_0 structures of complex **1**.

Center Number	Atomic Number	Atomic Type	Coordinates (Angstroms) X	Y	Z
1	79	0	0.011802	0.025754	0.050905
2	6	0	0.033738	-0.242844	2.099473
3	7	0	1.998046	-0.022517	0.334485
4	8	0	-8.679474	0.249951	-1.075167
5	6	0	-1.918426	0.077301	-0.225173
6	6	0	-3.129049	0.112220	-0.399538
7	6	0	-4.540640	0.152312	-0.598238
8	6	0	-5.430142	-0.067671	0.471986
9	1	0	-5.029683	-0.270935	1.461025

10	6	0	-6.799179	-0.028823	0.280546
11	1	0	-7.484991	-0.198882	1.105240
12	6	0	-7.329502	0.232917	-0.990429
13	6	0	-6.463536	0.454401	-2.065754
14	1	0	-6.846662	0.658823	-3.059449
15	6	0	-5.087967	0.412221	-1.863146
16	1	0	-4.421111	0.584999	-2.703099
17	6	0	-1.012383	-0.356589	3.011030
18	1	0	-2.038678	-0.305470	2.655946
19	6	0	-0.760610	-0.536252	4.373795
20	1	0	-1.590954	-0.623305	5.070352
21	6	0	0.549083	-0.604145	4.845197
22	1	0	0.743074	-0.743197	5.904909
23	6	0	1.613420	-0.493561	3.956000
24	1	0	2.631689	-0.546497	4.332694
25	6	0	1.365785	-0.314747	2.591340
26	6	0	2.443496	-0.192015	1.597493
27	6	0	3.820954	-0.230589	1.809461
28	1	0	4.222809	-0.366266	2.806660
29	6	0	4.671148	-0.091767	0.715878
30	1	0	5.745844	-0.119882	0.869039
31	6	0	4.168465	0.082529	-0.570647
32	1	0	4.839638	0.189803	-1.414635
33	6	0	2.786604	0.115559	-0.752513
34	6	0	2.036971	0.284407	-2.007085
35	6	0	2.664103	0.444002	-3.246782
36	1	0	3.748364	0.449082	-3.323670
37	6	0	1.897016	0.598370	-4.397287
38	1	0	2.385888	0.722570	-5.359352
39	6	0	0.506071	0.592944	-4.311609

40	1	0	-0.091390	0.713337	-5.212036
41	6	0	-0.124547	0.433636	-3.074856
42	1	0	-1.210433	0.430674	-3.021085
43	6	0	0.618681	0.277962	-1.907983
44	6	0	-9.260234	0.510918	-2.337847
45	1	0	-10.339677	0.480884	-2.184245
46	1	0	-8.977340	-0.251377	-3.074042
47	1	0	-8.974640	1.501256	-2.713003

Table S15. Optimized S_0 structures of complex **2**.

Center Number	Atomic Number	Atomic Type	Coordinates (Angstroms) X	Y	Z
1	79	0	-0.084471	0.321506	0.044532
2	7	0	-0.054457	0.273884	2.048074
3	6	0	1.922044	-0.080445	0.318458
4	6	0	2.934288	-0.253487	-0.591986
5	1	0	2.721484	-0.188753	-1.657421
6	6	0	4.272848	-0.517500	-0.188052
7	6	0	5.320497	-0.693836	-1.124292
8	1	0	5.090903	-0.626145	-2.185144
9	6	0	6.605769	-0.945688	-0.702927
10	1	0	7.400528	-1.078614	-1.431915
11	6	0	6.902571	-1.033054	0.676255
12	1	0	7.921781	-1.232120	0.995444
13	6	0	5.907198	-0.867048	1.609831
14	1	0	6.129771	-0.933063	2.672369
15	6	0	4.573692	-0.606155	1.205067
16	6	0	3.528790	-0.428332	2.142560

17	1	0	3.772115	-0.497161	3.200614
18	6	0	2.240030	-0.172662	1.724024
19	6	0	1.122763	0.026866	2.659678
20	6	0	1.152017	-0.016684	4.049639
21	1	0	2.077105	-0.245813	4.565689
22	6	0	-0.023007	0.202744	4.781191
23	6	0	-1.215154	0.459868	4.088009
24	1	0	-2.127929	0.660442	4.636871
25	6	0	-1.218299	0.489108	2.698485
26	6	0	-2.356109	0.737994	1.799000
27	6	0	-3.650721	0.985855	2.266228
28	1	0	-3.861685	1.004725	3.332429
29	6	0	-4.683547	1.210904	1.361913
30	1	0	-5.688401	1.403300	1.726976
31	1	0	-5.234483	1.364295	-0.712794
32	6	0	-4.425745	1.188511	-0.007581
33	6	0	-3.133948	0.940878	-0.478736
34	1	0	-2.945564	0.926048	-1.549401
35	6	0	-2.081477	0.712992	0.404157
36	6	0	-0.116728	0.366438	-1.905781
37	6	0	-0.139156	0.393530	-3.128904
38	6	0	-0.163855	0.423475	-4.554562
39	6	0	-1.345118	0.713848	-5.252708
40	1	0	-2.253463	0.919111	-4.693365
41	6	0	-1.379790	0.745191	-6.643016
42	1	0	-2.313464	0.974402	-7.144710
43	6	0	-0.215605	0.481899	-7.371297
44	6	0	0.973795	0.189759	-6.689433
45	1	0	1.869524	-0.012357	-7.269315
46	6	0	0.998167	0.161534	-5.306964

47	1	0	1.925334	-0.066024	-4.788808
48	8	0	-0.136490	0.485228	-8.721830
49	6	0	-1.311430	0.774964	-9.453352
50	1	0	-1.030106	0.724876	-10.506007
51	1	0	-2.099739	0.038978	-9.253212
52	1	0	-1.687339	1.780013	-9.225932
53	6	0	-0.007600	0.164229	6.258458
54	6	0	-1.097733	-0.355817	6.962974
55	6	0	1.096247	0.648421	6.967270
56	6	0	-1.102573	-0.402385	8.358387
57	1	0	-1.940304	-0.756997	6.406096
58	6	0	1.127127	0.625914	8.363038
59	1	0	1.928548	1.076454	6.414866
60	6	0	0.018412	0.095396	9.032472
61	1	0	0.028180	0.068936	10.114758
62	6	0	-2.311734	-0.990652	9.092033
63	6	0	-3.568977	-0.184663	8.725230
64	6	0	-2.503954	-2.455564	8.665995
65	6	0	-2.147142	-0.953190	10.613738
66	1	0	-3.463204	0.864070	9.025655
67	1	0	-3.769691	-0.208995	7.648729
68	1	0	-4.445317	-0.599535	9.237500
69	1	0	-1.624353	-3.057041	8.922495
70	1	0	-3.372480	-2.885522	9.179210
71	1	0	-2.673530	-2.549185	7.588008
72	1	0	-3.038733	-1.379598	11.086749
73	1	0	-1.283585	-1.540862	10.945997
74	1	0	-2.032356	0.070896	10.987390
75	6	0	2.350588	1.176380	9.102489
76	6	0	3.597568	0.382135	8.679752

77	6	0	2.543161	2.658080	8.739407
78	6	0	2.210448	1.071502	10.623467
79	1	0	3.492669	-0.677779	8.938542
80	1	0	3.778405	0.450438	7.601679
81	1	0	4.484816	0.772198	9.192755
82	1	0	1.669449	3.250586	9.033998
83	1	0	3.420027	3.061759	9.259697
84	1	0	2.699418	2.798969	7.664575
85	1	0	3.111234	1.473834	11.100221
86	1	0	1.354476	1.646320	10.995471
87	1	0	2.098442	0.032059	10.952840

Table S16. Optimized S_0 structures of complex **3-exo**.

Center Number	Atomic Number	Atomic Type	Coordinates (Angstroms) X	Y	Z
1	79	0	0.018801	0.099452	0.041414
2	7	0	0.017803	0.101186	2.043292
3	6	0	2.060191	0.059409	0.360590
4	6	0	0.025914	0.092290	-1.910309
5	6	0	-3.081496	0.169336	-0.542610
6	1	0	-2.872661	0.163059	-1.609646
7	6	0	-4.405377	0.200218	-0.096847
8	1	0	-5.219037	0.218013	-0.817915
9	1	0	-5.718255	0.232535	1.613110
10	6	0	-4.688594	0.208524	1.267742
11	6	0	-3.649424	0.185937	2.192753
12	1	0	-3.879786	0.193151	3.255088
13	6	0	-2.323226	0.154517	1.750978

14	6	0	-1.175607	0.129606	2.672861
15	6	0	-1.191101	0.123554	4.063071
16	1	0	-2.133958	0.117859	4.597882
17	6	0	0.017757	0.085714	4.773367
18	6	0	1.225420	0.058447	4.063254
19	1	0	2.167549	0.056827	4.599311
20	6	0	1.212780	0.067861	2.671365
21	6	0	2.360290	0.053598	1.754272
22	6	0	3.687549	0.039373	2.208291
23	1	0	3.910386	0.038055	3.273324
24	6	0	4.716050	0.030815	1.281608
25	6	0	4.428719	0.032708	-0.095087
26	6	0	3.108680	0.046765	-0.553739
27	1	0	2.892645	0.050654	-1.619722
28	6	0	5.695214	0.022159	-0.827493
29	6	0	5.956654	0.018142	-2.197442
30	1	0	5.145045	0.022590	-2.920848
31	6	0	7.282384	0.008261	-2.625791
32	1	0	7.505385	0.004959	-3.689321
33	6	0	8.328388	0.002552	-1.699530
34	1	0	9.356929	-0.005157	-2.050420
35	6	0	8.065498	0.006773	-0.327422
36	1	0	8.887796	0.002532	0.384700
37	6	0	6.746585	0.016638	0.106196
38	6	0	0.062996	0.072480	-4.559810
39	6	0	1.282688	0.037757	-5.264245
40	6	0	1.307695	0.025566	-6.646862
41	1	0	2.247929	-0.001391	-7.189654
42	6	0	0.111429	0.047663	-7.377155
43	6	0	-1.109762	0.082537	-6.697150

44	1	0	-2.050031	0.100361	-7.237178
45	6	0	-1.124236	0.094617	-5.306123
46	1	0	-2.076696	0.121598	-4.784447
47	1	0	2.215829	0.020166	-4.708316
48	6	0	0.039543	0.083689	-3.133929
49	6	0	6.214792	0.024987	1.529014
50	6	0	6.504532	-7.636690	2.205237
51	1	0	6.858242	-8.465106	2.829270
52	1	0	6.939400	-7.762353	1.206122
53	1	0	5.416590	-7.734540	2.105865
54	6	0	6.881305	-6.285761	2.801592
55	1	0	7.972460	-6.227563	2.921354
56	1	0	6.459502	-6.199695	3.813119
57	6	0	6.405982	-5.106362	1.958203
58	1	0	6.830435	-5.189616	0.946595
59	1	0	5.314311	-5.165414	1.834758
60	6	0	6.773819	-3.749362	2.550376
61	1	0	7.865506	-3.691174	2.674811
62	1	0	6.348082	-3.668023	3.561850
63	6	0	6.297140	-2.571854	1.704363
64	1	0	6.730524	-2.648560	0.697980
65	1	0	5.207697	-2.634302	1.576963
66	6	0	6.664964	-1.225411	2.319027
67	1	0	7.755623	-1.169707	2.446277
68	1	0	6.237906	-1.158604	3.330255
69	6	0	6.671396	1.283036	2.304637
70	1	0	6.235154	1.234854	3.312927
71	1	0	7.760449	1.218878	2.441498
72	6	0	6.321300	2.623537	1.666934
73	1	0	6.774508	2.686032	0.668318

74	1	0	5.234745	2.688659	1.517420
75	6	0	6.784238	3.810942	2.507276
76	1	0	6.334751	3.739407	3.507614
77	1	0	7.873157	3.751840	2.656267
78	6	0	6.939271	6.373164	2.652723
79	1	0	8.030820	6.304294	2.764926
80	1	0	6.751805	7.274954	2.054764
81	6	0	6.437189	5.156357	1.874270
82	1	0	5.345734	5.229012	1.754518
83	1	0	6.856833	5.185792	0.858726
84	6	0	6.296151	6.547059	4.024976
85	1	0	6.645051	7.465096	4.511091
86	1	0	6.529892	5.712115	4.694949
87	1	0	5.203940	6.608966	3.940680
88	6	0	-2.022652	0.145831	0.361181
89	8	0	0.241044	0.032949	-8.723733
90	6	0	-0.939214	0.051974	-9.502409
91	1	0	-1.524403	0.961653	-9.320421
92	1	0	-0.613115	0.035019	-10.543126
93	1	0	-1.564089	-0.827292	-9.303637
94	6	0	0.018097	0.073485	6.251255
95	6	0	0.934373	-0.717929	6.949956
96	6	0	-0.897818	0.852355	6.964277
97	6	0	0.947483	-0.748209	8.345959
98	1	0	1.623754	-1.341942	6.387354
99	6	0	-0.909526	0.858437	8.360673
100	1	0	-1.588578	1.485166	6.413311
101	6	0	0.019391	0.049448	9.025462
102	1	0	0.019913	0.040030	10.108238
103	6	0	-1.912125	1.745366	9.105251

104	6	0	-1.645288	3.217426	8.749333
105	6	0	-3.341673	1.370648	8.681083
106	6	0	-1.810232	1.593162	10.625325
107	1	0	-0.634105	3.515963	9.048776
108	1	0	-1.746493	3.400810	7.674218
109	1	0	-2.360350	3.866818	9.268621
110	1	0	-3.562706	0.325807	8.927485
111	1	0	-4.067674	2.005149	9.203403
112	1	0	-3.497000	1.504356	7.605238
113	1	0	-2.551688	2.240949	11.106107
114	1	0	-2.010546	0.564430	10.946670
115	1	0	-0.822950	1.885827	11.000842
116	6	0	1.949764	-1.649150	9.074040
117	6	0	1.678117	-3.114653	8.695216
118	6	0	3.379356	-1.271794	8.652408
119	6	0	1.851964	-1.520661	10.596521
120	1	0	0.667021	-3.415357	8.992751
121	1	0	1.775551	-3.280592	7.616929
122	1	0	2.392956	-3.774363	9.201703
123	1	0	3.603709	-0.231310	8.913771
124	1	0	4.104803	-1.915861	9.163676
125	1	0	3.532043	-1.390139	7.574370
126	1	0	2.593558	-2.177115	11.065161
127	1	0	2.054737	-0.497304	10.933130
128	1	0	0.865175	-1.817485	10.970028

Table S17. Optimized S_0 structures of complex **3-endo**.

Center Number	Atomic Number	Atomic Type	Coordinates (Angstroms) X	Y	Z

1	79	0	-0.008708	-0.097263	0.000965
2	7	0	-0.009251	-0.119995	2.009559
3	6	0	2.100433	-0.068407	0.338541
4	6	0	-0.272270	-0.055309	-1.934969
5	8	0	-1.846117	0.279886	-8.558806
6	6	0	-2.048085	-0.148422	0.333096
7	6	0	-3.103660	-0.161868	-0.574312
8	1	0	-2.892441	-0.140405	-1.639370
9	6	0	-4.427488	-0.201325	-0.130253
10	1	0	-5.238243	-0.210965	-0.854635
11	6	0	-4.714848	-0.227913	1.232836
12	1	0	-5.744977	-0.258405	1.576125
13	6	0	-3.677017	-0.214784	2.158150
14	1	0	-3.907205	-0.235753	3.220072
15	6	0	-2.349960	-0.174930	1.718770
16	6	0	-1.205976	-0.155916	2.637328
17	6	0	-1.235688	-0.159506	4.025144
18	1	0	-2.184078	-0.161603	4.549747
19	6	0	-0.034059	-0.120492	4.746564
20	6	0	1.174197	-0.080202	4.043620
21	1	0	2.109400	-0.077310	4.589937
22	6	0	1.173592	-0.081214	2.648877
23	6	0	-0.044941	-0.121907	6.224281
24	6	0	-0.964962	-0.909821	6.921941
25	1	0	-1.650317	-1.537993	6.359013
26	6	0	-0.987525	-0.930726	8.318028
27	6	0	-0.065301	-0.127106	8.998481
28	1	0	-0.073379	-0.129129	10.081270
29	6	0	0.866489	0.679366	8.334635

30	6	0	0.864127	0.664028	6.938309
31	1	0	1.556609	1.295346	6.387835
32	6	0	2.335242	-0.059157	1.754522
33	6	0	3.622251	-0.041499	2.307621
34	1	0	3.756570	-0.030881	3.385020
35	6	0	4.738371	-0.042988	1.491178
36	1	0	5.737613	-0.032922	1.917766
37	6	0	4.542895	-0.063586	0.115201
38	6	0	5.555802	-0.083968	-0.933354
39	6	0	6.949393	-0.088464	-0.855867
40	1	0	7.458221	-0.072612	0.104886
41	6	0	7.682668	-0.114764	-2.039170
42	1	0	8.768740	-0.119115	-2.001805
43	6	0	7.031745	-0.136242	-3.276493
44	1	0	7.618274	-0.157223	-4.191325
45	6	0	5.637452	-0.131330	-3.348827
46	1	0	5.141702	-0.148175	-4.316999
47	6	0	4.901666	-0.105143	-2.170164
48	6	0	3.395862	-0.090841	-1.992247
49	6	0	3.247662	-0.072016	-0.464557
50	6	0	-0.602485	-0.014305	-3.112177
51	6	0	-0.934224	0.039564	-4.498223
52	6	0	-1.827268	-0.881252	-5.064821
53	1	0	-2.270154	-1.646900	-4.434103
54	6	0	-2.155791	-0.838327	-6.416285
55	1	0	-2.848098	-1.571096	-6.816098
56	6	0	-1.593839	0.144968	-7.235710
57	6	0	-0.701983	1.074869	-6.684029
58	1	0	-0.273766	1.833101	-7.333052
59	6	0	-0.376092	1.021233	-5.340711

60	1	0	0.318229	1.746015	-4.925257
61	6	0	-2.742173	-0.635641	-9.156833
62	1	0	-3.739923	-0.572682	-8.705295
63	1	0	-2.373779	-1.665962	-9.079160
64	1	0	-2.803752	-0.353086	-10.208588
65	6	0	-1.994277	-1.827279	9.045300
66	6	0	1.861842	1.573823	9.079965
67	6	0	3.295191	1.201613	8.666542
68	1	0	3.518148	0.158791	8.919647
69	1	0	4.015841	1.841183	9.190092
70	1	0	3.456840	1.330426	7.591021
71	6	0	1.751443	1.430025	10.600266
72	1	0	0.761153	1.721890	10.968377
73	1	0	2.488191	2.082740	11.081622
74	1	0	1.952881	0.403700	10.928562
75	6	0	1.592159	3.042873	8.713822
76	1	0	1.698775	3.220093	7.638204
77	1	0	2.302102	3.697797	9.233210
78	1	0	0.578297	3.339742	9.005783
79	6	0	-3.421139	-1.450483	8.614008
80	1	0	-3.645912	-0.408627	8.869488
81	1	0	-4.150109	-2.091582	9.124004
82	1	0	-3.567822	-1.573289	7.535653
83	6	0	-1.904378	-1.691221	10.567601
84	1	0	-0.919559	-1.986413	10.947606
85	1	0	-2.648561	-2.345142	11.035681
86	1	0	-2.108618	-0.666194	10.898152
87	6	0	-1.722204	-3.294978	8.675476
88	1	0	-1.814570	-3.466605	7.597634
89	1	0	-2.440282	-3.951221	9.181864

90	1	0	-0.712920	-3.595181	8.979605
91	6	0	2.776632	-1.354342	-2.631587
92	1	0	1.690654	-1.303744	-2.507122
93	1	0	2.963858	-1.303477	-3.714617
94	6	0	3.289872	-2.688022	-2.097237
95	1	0	4.378032	-2.756515	-2.234717
96	1	0	3.111257	-2.744775	-1.014335
97	6	0	2.620617	-3.879474	-2.777190
98	1	0	2.789031	-3.821649	-3.863164
99	1	0	1.531298	-3.814008	-2.635156
100	6	0	3.114435	-5.228353	-2.263381
101	1	0	4.203197	-5.296655	-2.407853
102	1	0	2.948037	-5.287080	-1.177175
103	6	0	2.441816	-6.419329	-2.939627
104	1	0	2.607180	-6.359047	-4.024735
105	1	0	1.354462	-6.350202	-2.793732
106	6	0	2.942111	-7.762212	-2.420782
107	1	0	4.021446	-7.870591	-2.583654
108	1	0	2.443476	-8.599436	-2.922110
109	1	0	2.759525	-7.861528	-1.343745
110	6	0	2.805471	1.174781	-2.655929
111	1	0	2.988124	1.095746	-3.738265
112	1	0	1.719575	1.152425	-2.526128
113	6	0	3.350935	2.507808	-2.152887
114	1	0	3.169940	2.596051	-1.072557
115	1	0	4.440804	2.546243	-2.287791
116	6	0	2.713427	3.698074	-2.864928
117	1	0	1.621656	3.659557	-2.731178
118	1	0	2.890657	3.613004	-3.947935
119	6	0	3.232064	5.047011	-2.376088

120	1	0	3.055969	5.133037	-1.293294
121	1	0	4.323455	5.087891	-2.510462
122	6	0	2.593189	6.237529	-3.084995
123	1	0	1.502997	6.194617	-2.950921
124	1	0	2.770087	6.151481	-4.166547
125	6	0	3.116371	7.579813	-2.587578
126	1	0	2.924081	7.704752	-1.514932
127	1	0	2.641266	8.416974	-3.111310
128	1	0	4.199506	7.662096	-2.740265

Table S18. Optimized ^{3}IL state of complex **1**.

Center Number	Atomic Number	Atomic Type	Coordinates (Angstroms) X	Y	Z
1	79	0	-0.008307	0.009540	-0.001996
2	6	0	-0.006560	-0.012111	2.061108
3	7	0	1.952548	-0.003598	0.314321
4	8	0	-8.695390	0.088496	-1.236058
5	6	0	-1.940565	0.023746	-0.307881
6	6	0	-3.150029	0.034213	-0.496730
7	6	0	-4.559625	0.047220	-0.712028
8	6	0	-5.459652	0.044541	0.372173
9	1	0	-5.068777	0.031641	1.385575
10	6	0	-6.826906	0.058347	0.164432
11	1	0	-7.520047	0.056502	1.000512
12	6	0	-7.345812	0.075408	-1.137594
13	6	0	-6.469898	0.077893	-2.227301
14	1	0	-6.843966	0.090641	-3.245076

15	6	0	-5.096165	0.063965	-2.008010
16	1	0	-4.421785	0.066211	-2.859695
17	6	0	-1.058691	-0.016764	2.974647
18	1	0	-2.082996	-0.008436	2.610601
19	6	0	-0.813782	-0.032160	4.348939
20	1	0	-1.648492	-0.035536	5.045783
21	6	0	0.494325	-0.043380	4.838743
22	1	0	0.676485	-0.055366	5.909382
23	6	0	1.564458	-0.039224	3.954230
24	1	0	2.579787	-0.048045	4.341360
25	6	0	1.325490	-0.023712	2.573568
26	6	0	2.394885	-0.018485	1.574993
27	6	0	3.781795	-0.027364	1.813463
28	1	0	4.161267	-0.038940	2.827966
29	6	0	4.678155	-0.021064	0.710594
30	1	0	5.747247	-0.028650	0.894442
31	6	0	4.199576	-0.005618	-0.573205
32	1	0	4.882200	-0.001241	-1.416127
33	6	0	2.783860	0.004439	-0.812610
34	6	0	2.098244	0.021405	-2.024581
35	6	0	2.737215	0.033081	-3.317571
36	1	0	3.821033	0.029553	-3.387259
37	6	0	1.977331	0.048871	-4.450418
38	1	0	2.459817	0.057646	-5.423878
39	6	0	0.551800	0.054256	-4.381179
40	1	0	-0.027437	0.066815	-5.299713
41	6	0	-0.096264	0.043664	-3.137461
42	1	0	-1.182936	0.048219	-3.102285
43	6	0	0.615588	0.027596	-1.952816
44	6	0	-9.264285	0.107296	-2.530324

45	1	0	-10.345094	0.116957	-2.383620
46	1	0	-8.984419	-0.784566	-3.104443
47	1	0	-8.965922	1.004506	-3.086493

Table S19. Optimized ^{3}IL state of complex **2**.

Center Number	Atomic Number	Atomic Type	Coordinates (Angstroms) X	Y	Z
1	79	0	-0.064775	0.018658	0.006848
2	7	0	-0.062408	0.020342	1.998853
3	6	0	1.979370	-0.013737	0.332691
4	6	0	3.025655	-0.036032	-0.568605
5	1	0	2.813286	-0.036340	-1.636018
6	6	0	4.413647	-0.060133	-0.158471
7	6	0	5.445391	-0.084657	-1.077850
8	1	0	5.210420	-0.086362	-2.139674
9	6	0	6.805033	-0.107543	-0.662688
10	1	0	7.594392	-0.126653	-1.407406
11	6	0	7.104919	-0.104985	0.686265
12	1	0	8.139229	-0.122200	1.017613
13	6	0	6.075727	-0.080262	1.639633
14	1	0	6.317763	-0.078747	2.699376
15	6	0	4.714987	-0.057790	1.247281
16	6	0	3.666413	-0.033602	2.173272
17	1	0	3.898742	-0.029659	3.234232
18	6	0	2.266880	-0.013551	1.729360
19	6	0	1.160692	0.006469	2.621378
20	6	0	1.170635	0.004295	4.031829
21	1	0	2.114733	-0.038485	4.563363

22	6	0	-0.026093	0.016682	4.742282
23	6	0	-1.250571	0.030596	4.034584
24	1	0	-2.189058	0.072487	4.574577
25	6	0	-1.241559	0.032250	2.642470
26	6	0	-2.397292	0.052916	1.728619
27	6	0	-3.721424	0.073098	2.174824
28	1	0	-3.946413	0.074258	3.238338
29	6	0	-4.765064	0.092278	1.253707
30	1	0	-5.793482	0.108441	1.603362
31	1	0	-5.304174	0.105753	-0.829553
32	6	0	-4.487662	0.090810	-0.111625
33	6	0	-3.164869	0.069986	-0.563205
34	1	0	-2.960416	0.068667	-1.631084
35	6	0	-2.103387	0.050949	0.336825
36	6	0	-0.056558	0.016415	-1.947883
37	6	0	-0.035049	0.013899	-3.172092
38	6	0	-0.005027	0.010990	-4.597414
39	6	0	-1.188992	0.037225	-5.349266
40	1	0	-2.143803	0.060167	-4.831762
41	6	0	-1.168016	0.034537	-6.740119
42	1	0	-2.105764	0.055417	-7.284397
43	6	0	0.056436	0.005045	-7.414750
44	6	0	1.249427	-0.021392	-6.679063
45	1	0	2.192116	-0.044183	-7.217774
46	6	0	1.218027	-0.018424	-5.296641
47	1	0	2.148505	-0.039117	-4.736512
48	8	0	0.192315	-0.000319	-8.760567
49	6	0	-0.984233	0.023718	-9.544889
50	1	0	-0.653000	0.013879	-10.584060
51	1	0	-1.609755	-0.857018	-9.355047

52	1	0	-1.570437	0.932034	-9.359535
53	6	0	-0.022297	0.008481	6.220767
54	6	0	-0.979460	-0.727506	6.925988
55	6	0	0.937773	0.734617	6.932292
56	6	0	-0.989761	-0.755520	8.322250
57	1	0	-1.707230	-1.310257	6.367402
58	6	0	0.959256	0.736086	8.328528
59	1	0	1.658784	1.330182	6.378402
60	6	0	-0.012541	-0.016260	8.998571
61	1	0	-0.009042	-0.025474	10.081324
62	6	0	-2.043986	-1.591834	9.054244
63	6	0	-3.448363	-1.129433	8.632428
64	6	0	-1.862263	-3.072510	8.681073
65	6	0	-1.938328	-1.463979	10.576445
66	1	0	-3.606742	-0.075275	8.887189
67	1	0	-3.609939	-1.244831	7.555377
68	1	0	-4.211797	-1.723493	9.149032
69	1	0	-0.871730	-3.432998	8.981590
70	1	0	-2.616844	-3.685566	9.188629
71	1	0	-1.967541	-3.235793	7.603139
72	1	0	-2.718316	-2.072590	11.047538
73	1	0	-0.971398	-1.818355	10.951758
74	1	0	-2.079161	-0.428954	10.909066
75	6	0	2.017735	1.560346	9.068014
76	6	0	3.419463	1.107754	8.627196
77	6	0	1.831640	3.047493	8.724111
78	6	0	1.923574	1.404170	10.588347
79	1	0	3.581568	0.049657	8.862642
80	1	0	3.572062	1.241677	7.550981
81	1	0	4.186090	1.694257	9.147686

82	1	0	0.842724	3.401080	9.037775
83	1	0	2.588876	3.651695	9.238275
84	1	0	1.929676	3.231403	7.648806
85	1	0	2.706650	2.004450	11.065008
86	1	0	0.959252	1.750995	10.977188
87	1	0	2.067151	0.363313	10.901031

Table S20. Optimized ^{3}IL state of complex **3-exo**.

Center Number	Atomic Number	Atomic Type	Coordinates (Angstroms) X	Y	Z
1	79	0	0.014062	-0.018325	-0.011838
2	7	0	0.013628	-0.026455	1.975212
3	6	0	2.045165	-0.016403	0.292460
4	6	0	0.013952	-0.009457	-1.968938
5	6	0	-3.084980	-0.015835	-0.591285
6	1	0	-2.876826	-0.014137	-1.658516
7	6	0	-4.407593	-0.015531	-0.144027
8	1	0	-5.222484	-0.013804	-0.864068
9	1	0	-5.720356	-0.016834	1.567556
10	6	0	-4.690364	-0.017112	1.222113
11	6	0	-3.651107	-0.019134	2.145870
12	1	0	-3.880127	-0.020000	3.208572
13	6	0	-2.323842	-0.020176	1.703565
14	6	0	-1.171281	-0.021812	2.616193
15	6	0	-1.187898	-0.025331	4.006480
16	1	0	-2.132732	-0.052103	4.536819
17	6	0	0.032231	-0.037359	4.736316
18	6	0	1.228259	-0.040628	4.033652

19	1	0	2.169853	-0.019808	4.571721
20	6	0	1.235983	-0.033639	2.620183
21	6	0	2.349068	-0.022688	1.751758
22	6	0	3.714058	-0.014225	2.203255
23	1	0	3.937899	-0.015101	3.267108
24	6	0	4.712874	-0.000388	1.283823
25	6	0	4.423098	0.000749	-0.142294
26	6	0	3.079378	-0.006908	-0.601807
27	1	0	2.869829	-0.003005	-1.669095
28	6	0	5.653743	0.013537	-0.849079
29	6	0	5.915022	0.016946	-2.236987
30	1	0	5.098155	0.009025	-2.953192
31	6	0	7.231006	0.030030	-2.665893
32	1	0	7.452404	0.032603	-3.729414
33	6	0	8.288378	0.039992	-1.741185
34	1	0	9.313303	0.050233	-2.100776
35	6	0	8.037663	0.037028	-0.361710
36	1	0	8.867772	0.045399	0.340519
37	6	0	6.729849	0.023797	0.083815
38	6	0	0.032940	0.008743	-4.619222
39	6	0	1.246388	0.037874	-5.334809
40	6	0	1.259976	0.048630	-6.717822
41	1	0	2.195929	0.071333	-7.268270
42	6	0	0.057962	0.030386	-7.438338
43	6	0	-1.157253	0.001059	-6.747821
44	1	0	-2.102236	-0.013764	-7.279742
45	6	0	-1.160186	-0.009342	-5.356524
46	1	0	-2.108569	-0.031957	-4.827224
47	1	0	2.184482	0.052307	-4.787083
48	6	0	0.020723	-0.001363	-3.193093

49	6	0	6.214609	0.020132	1.513762
50	6	0	6.631569	-7.636955	2.113423
51	1	0	6.996763	-8.466050	2.729849
52	1	0	7.072790	-7.744029	1.114948
53	1	0	5.545903	-7.752242	2.008259
54	6	0	6.982659	-6.286598	2.726524
55	1	0	8.072150	-6.210939	2.851528
56	1	0	6.555220	-6.219151	3.737048
57	6	0	6.490370	-5.106334	1.894151
58	1	0	6.918763	-5.171931	0.882927
59	1	0	5.400132	-5.181655	1.767383
60	6	0	6.835213	-3.750115	2.501761
61	1	0	7.925693	-3.674478	2.626983
62	1	0	6.407803	-3.686530	3.513633
63	6	0	6.338956	-2.571848	1.668483
64	1	0	6.768645	-2.634036	0.659176
65	1	0	5.249377	-2.647894	1.546370
66	6	0	6.691402	-1.226064	2.293416
67	1	0	7.781916	-1.152562	2.412149
68	1	0	6.269339	-1.171982	3.307078
69	6	0	6.652075	1.287151	2.284179
70	1	0	6.227118	1.228817	3.296373
71	1	0	7.743745	1.247078	2.407293
72	6	0	6.261728	2.616124	1.646014
73	1	0	6.697279	2.684600	0.639624
74	1	0	5.171335	2.655585	1.514243
75	6	0	6.710178	3.817854	2.473363
76	1	0	6.278745	3.740011	3.481041
77	1	0	7.802415	3.784671	2.604530
78	6	0	6.806351	6.383148	2.605270

79	1	0	7.900738	6.340107	2.701433
80	1	0	6.589163	7.277297	2.005951
81	6	0	6.321038	5.151386	1.839861
82	1	0	5.226451	5.197480	1.737311
83	1	0	6.723875	5.186069	0.817690
84	6	0	6.179603	6.548793	3.986068
85	1	0	6.514981	7.476598	4.463007
86	1	0	6.441741	5.722420	4.656191
87	1	0	5.085205	6.585958	3.917547
88	6	0	-2.023639	-0.018355	0.311913
89	8	0	0.176467	0.043003	-8.786652
90	6	0	-1.010534	0.027314	-9.554605
91	1	0	-1.630744	0.908973	-9.351487
92	1	0	-0.693904	0.041858	-10.598329
93	1	0	-1.597594	-0.879979	-9.366421
94	6	0	0.018723	-0.037998	6.213300
95	6	0	0.963263	-0.777376	6.933009
96	6	0	-0.938730	0.703216	6.913633
97	6	0	0.971577	-0.784035	8.329211
98	1	0	1.682423	-1.380277	6.384517
99	6	0	-0.963251	0.724674	8.309876
100	1	0	-1.655630	1.296257	6.351578
101	6	0	0.000293	-0.025761	8.993498
102	1	0	-0.007496	-0.021693	10.076229
103	6	0	-2.016754	1.568195	9.034843
104	6	0	-1.815867	3.048792	8.671149
105	6	0	-3.421366	1.122798	8.596122
106	6	0	-1.928508	1.432115	10.557429
107	1	0	-0.824553	3.397379	8.982955
108	1	0	-1.908882	3.218524	7.593077

109	1	0	-2.568819	3.667372	9.174461
110	1	0	-3.594043	0.069571	8.845448
111	1	0	-4.183927	1.723467	9.106360
112	1	0	-3.569800	1.243440	7.517788
113	1	0	-2.707483	2.045907	11.023484
114	1	0	-2.082378	0.396869	10.883495
115	1	0	-0.962154	1.775319	10.944478
116	6	0	2.013400	-1.623574	9.075442
117	6	0	1.809737	-3.107496	8.727360
118	6	0	3.424805	-1.189831	8.646884
119	6	0	1.909542	-1.468955	10.595322
120	1	0	0.813392	-3.447795	9.032085
121	1	0	1.913951	-3.290700	7.652503
122	1	0	2.554083	-3.723316	9.246624
123	1	0	3.599813	-0.134552	8.885785
124	1	0	4.178979	-1.787999	9.172328
125	1	0	3.584195	-1.323776	7.571653
126	1	0	2.681639	-2.079478	11.076909
127	1	0	2.063040	-0.430356	10.910810
128	1	0	0.938163	-1.804767	10.976094

Table S21. Optimized ^{3}IL state of complex **3-endo**.

Center Number	Atomic Number	Atomic Type	Coordinates (Angstroms) X	Y	Z
1	79	0	-0.009426	0.022733	-0.019773
2	7	0	-0.000964	0.015765	1.974036
3	6	0	2.086980	0.060012	0.298247
4	6	0	-0.282295	0.055384	-1.959049

5	8	0	-2.052723	0.257162	-8.541897
6	6	0	-2.043829	-0.039108	0.326712
7	6	0	-3.106802	-0.072070	-0.573417
8	1	0	-2.902911	-0.059343	-1.640149
9	6	0	-4.426372	-0.120947	-0.120262
10	1	0	-5.241891	-0.145087	-0.839135
11	6	0	-4.705661	-0.139694	1.245928
12	1	0	-5.733571	-0.178158	1.595233
13	6	0	-3.662806	-0.109838	2.163938
14	1	0	-3.885361	-0.127151	3.227651
15	6	0	-2.338103	-0.059512	1.715950
16	6	0	-1.184659	-0.026932	2.619052
17	6	0	-1.208282	-0.027195	4.008199
18	1	0	-2.156747	-0.031489	4.532435
19	6	0	0.005158	0.022745	4.742909
20	6	0	1.199416	0.072125	4.040030
21	1	0	2.136541	0.079940	4.584453
22	6	0	1.210516	0.069856	2.625731
23	6	0	-0.011695	0.013165	6.219394
24	6	0	-0.946156	-0.764351	6.911449
25	1	0	-1.640592	-1.377396	6.342695
26	6	0	-0.975038	-0.795753	8.307348
27	6	0	-0.040548	-0.016817	8.999472
28	1	0	-0.052596	-0.027707	10.082134
29	6	0	0.906819	0.778245	8.343631
30	6	0	0.904715	0.779341	6.947380
31	1	0	1.605618	1.409208	6.405606
32	6	0	2.330038	0.104064	1.768057
33	6	0	3.665221	0.172635	2.321501
34	1	0	3.795528	0.240255	3.396422

35	6	0	4.749037	0.146728	1.513792
36	1	0	5.754369	0.191458	1.922638
37	6	0	4.546733	0.060590	0.099241
38	6	0	5.533317	0.002452	-0.911047
39	6	0	6.944410	-0.002480	-0.824383
40	1	0	7.443648	0.043859	0.139353
41	6	0	7.677551	-0.070219	-1.995830
42	1	0	8.763151	-0.076224	-1.954939
43	6	0	7.033555	-0.132401	-3.243763
44	1	0	7.630017	-0.185161	-4.150173
45	6	0	5.634140	-0.127162	-3.333956
46	1	0	5.152610	-0.174788	-4.307693
47	6	0	4.887520	-0.060248	-2.173163
48	6	0	3.380937	-0.033036	-2.008489
49	6	0	3.218689	0.032351	-0.482292
50	6	0	-0.616071	0.081337	-3.136420
51	6	0	-0.959047	0.112044	-4.520556
52	6	0	-0.196007	-0.578572	-5.473586
53	1	0	0.671675	-1.144705	-5.147207
54	6	0	-0.527524	-0.558371	-6.824884
55	1	0	0.086921	-1.108437	-7.529218
56	6	0	-1.641747	0.168201	-7.254978
57	6	0	-2.413076	0.866918	-6.316423
58	1	0	-3.275429	1.427241	-6.665636
59	6	0	-2.078811	0.836743	-4.974427
60	1	0	-2.685095	1.381282	-4.256053
61	6	0	-1.308344	-0.436853	-9.522943
62	1	0	-1.308999	-1.517838	-9.336229
63	1	0	-0.272817	-0.077797	-9.569433
64	1	0	-1.802517	-0.235198	-10.474266

65	6	0	-2.001156	-1.680209	9.022828
66	6	0	1.915815	1.648932	9.099260
67	6	0	3.343510	1.262064	8.679950
68	1	0	3.551695	0.213000	8.919680
69	1	0	4.074002	1.884590	9.210562
70	1	0	3.505534	1.401630	7.605844
71	6	0	1.805638	1.487518	10.617929
72	1	0	0.820685	1.790274	10.991493
73	1	0	2.553437	2.122091	11.106682
74	1	0	1.990769	0.453863	10.932630
75	6	0	1.667198	3.126473	8.753128
76	1	0	1.775021	3.316151	7.679755
77	1	0	2.386655	3.764630	9.280444
78	1	0	0.657775	3.433654	9.049865
79	6	0	-3.419171	-1.279387	8.584313
80	1	0	-3.629099	-0.235284	8.843324
81	1	0	-4.161704	-1.911239	9.086392
82	1	0	-3.560479	-1.394490	7.504430
83	6	0	-1.920444	-1.554019	10.546562
84	1	0	-0.943589	-1.867583	10.932416
85	1	0	-2.679042	-2.197648	11.005882
86	1	0	-2.109491	-0.527252	10.880788
87	6	0	-1.749645	-3.150130	8.647208
88	1	0	-1.836017	-3.313921	7.567668
89	1	0	-2.481525	-3.798293	9.144385
90	1	0	-0.747347	-3.467517	8.957151
91	6	0	2.762719	-1.313610	-2.610965
92	1	0	1.676550	-1.255263	-2.492119
93	1	0	2.958125	-1.297230	-3.694011
94	6	0	3.273253	-2.628401	-2.029478

95	1	0	4.362317	-2.701284	-2.159884
96	1	0	3.088793	-2.647115	-0.946141
97	6	0	2.607625	-3.842712	-2.671196
98	1	0	2.775155	-3.818052	-3.758702
99	1	0	1.518451	-3.776799	-2.529646
100	6	0	3.108055	-5.172627	-2.116065
101	1	0	4.197057	-5.240088	-2.259279
102	1	0	2.942395	-5.198282	-1.028593
103	6	0	2.440734	-6.387198	-2.754559
104	1	0	2.603528	-6.358840	-3.841381
105	1	0	1.353404	-6.319933	-2.608245
106	6	0	2.950040	-7.710759	-2.196396
107	1	0	4.029721	-7.817722	-2.357958
108	1	0	2.455425	-8.565502	-2.671371
109	1	0	2.769715	-7.778751	-1.116585
110	6	0	2.805851	1.218453	-2.709676
111	1	0	3.000326	1.113465	-3.787566
112	1	0	1.718732	1.204206	-2.588573
113	6	0	3.357621	2.556292	-2.226727
114	1	0	3.162217	2.666658	-1.150743
115	1	0	4.449976	2.582491	-2.347781
116	6	0	2.741182	3.738792	-2.969150
117	1	0	1.647619	3.710807	-2.851361
118	1	0	2.933399	3.633315	-4.047483
119	6	0	3.264091	5.091091	-2.494549
120	1	0	3.072972	5.196151	-1.415985
121	1	0	4.357660	5.121649	-2.613685
122	6	0	2.644845	6.274880	-3.231638
123	1	0	1.552545	6.241671	-3.113394
124	1	0	2.837199	6.170635	-4.308903

125	6	0	3.170227	7.621050	-2.747210
126	1	0	2.962807	7.764028	-1.679657
127	1	0	2.708914	8.453176	-3.290903
128	1	0	4.256089	7.693648	-2.884894

Table S22. Optimized ^{3}LLCT state of complex **1**.

Center Number	Atomic Number	Atomic Type	Coordinates (Angstroms) X	Y	Z
1	79	0	-0.018347	-0.024757	-0.022465
2	6	0	-0.017241	0.153699	2.044697
3	7	0	1.933000	0.002689	0.308858
4	8	0	-8.558832	-0.334461	-1.525588
5	6	0	-1.911320	-0.059789	-0.345502
6	6	0	-3.137214	-0.086833	-0.559892
7	6	0	-4.503741	-0.125340	-0.802177
8	6	0	-5.211719	-1.363174	-0.793789
9	1	0	-4.665691	-2.278736	-0.592238
10	6	0	-6.558113	-1.394942	-1.036750
11	1	0	-7.114942	-2.326064	-1.035140
12	6	0	-7.260936	-0.193639	-1.300969
13	6	0	-6.579968	1.043535	-1.313127
14	1	0	-7.109737	1.967286	-1.513366
15	6	0	-5.227524	1.070790	-1.067178
16	1	0	-4.691992	2.014463	-1.073533
17	6	0	-1.107025	0.229838	2.915633
18	1	0	-2.119864	0.197420	2.523453
19	6	0	-0.909598	0.348175	4.290446

20	1	0	-1.766781	0.406598	4.956811
21	6	0	0.389219	0.391640	4.810660
22	1	0	0.543447	0.483795	5.882606
23	6	0	1.483453	0.317327	3.963186
24	1	0	2.487802	0.351521	4.379046
25	6	0	1.304388	0.197942	2.573482
26	6	0	2.385680	0.115089	1.612742
27	6	0	3.749608	0.135043	1.843809
28	1	0	4.120718	0.221844	2.860305
29	6	0	4.647257	0.043411	0.769603
30	1	0	5.717015	0.059193	0.951119
31	6	0	4.157222	-0.068291	-0.540171
32	1	0	4.845555	-0.138995	-1.376718
33	6	0	2.793470	-0.088606	-0.772340
34	6	0	2.092538	-0.195680	-2.035757
35	6	0	2.723390	-0.301875	-3.288097
36	1	0	3.809236	-0.308806	-3.349127
37	6	0	1.972754	-0.397775	-4.449325
38	1	0	2.474835	-0.479191	-5.409870
39	6	0	0.574386	-0.389985	-4.387581
40	1	0	-0.012818	-0.465108	-5.299584
41	6	0	-0.068827	-0.286252	-3.155319
42	1	0	-1.154806	-0.281528	-3.119530
43	6	0	0.669768	-0.188790	-1.973466
44	6	0	-9.356093	0.814839	-1.805453
45	1	0	-10.367345	0.437724	-1.949755
46	1	0	-9.011497	1.310829	-2.717709
47	1	0	-9.337305	1.512715	-0.963078

Table S23. Optimized ^{3}LLCT state of complex **3-endo**.

Center Number	Atomic Number	Atomic Type	Coordinates (Angstroms) X	Y	Z
1	79	0	0.015212	-0.018864	-0.007959
2	7	0	0.009539	-0.014986	1.969964
3	6	0	2.114780	-0.000814	0.357798
4	6	0	-0.248915	-0.017403	-1.913068
5	8	0	-2.147165	0.023195	-8.395920
6	6	0	-2.038704	-0.048332	0.338203
7	6	0	-3.091132	-0.055468	-0.580920
8	1	0	-2.886553	-0.048639	-1.647016
9	6	0	-4.415563	-0.070693	-0.147857
10	1	0	-5.221017	-0.076062	-0.878117
11	6	0	-4.706750	-0.078180	1.219433
12	1	0	-5.739116	-0.088392	1.558672
13	6	0	-3.676985	-0.071403	2.147165
14	1	0	-3.912069	-0.076177	3.208661
15	6	0	-2.336293	-0.057251	1.728045
16	6	0	-1.202159	-0.054626	2.632652
17	6	0	-1.220980	-0.084195	4.009368
18	1	0	-2.176521	-0.087394	4.523297
19	6	0	-0.020198	-0.066562	4.757531
20	6	0	1.191439	-0.021386	4.033551
21	1	0	2.133148	-0.044560	4.570874
22	6	0	1.205001	-0.002828	2.651924
23	6	0	-0.034027	-0.106266	6.225792
24	6	0	-1.067137	-0.755133	6.919803
25	1	0	-1.844696	-1.255184	6.348371
26	6	0	-1.097381	-0.811653	8.313660

27	6	0	-0.056696	-0.198500	9.021850
28	1	0	-0.064404	-0.236052	10.103768
29	6	0	0.993921	0.461511	8.372311
30	6	0	0.985828	0.498019	6.977330
31	1	0	1.771600	1.034867	6.452382
32	6	0	2.356231	0.012791	1.773303
33	6	0	3.663742	0.038402	2.291012
34	1	0	3.816461	0.055128	3.366527
35	6	0	4.758090	0.041762	1.452318
36	1	0	5.766423	0.059184	1.857913
37	6	0	4.540684	0.020513	0.073812
38	6	0	5.533518	0.012477	-0.991999
39	6	0	6.928874	0.023326	-0.943191
40	1	0	7.455824	0.041975	0.007788
41	6	0	7.641103	0.009455	-2.140817
42	1	0	8.727886	0.017457	-2.121351
43	6	0	6.971151	-0.014661	-3.366647
44	1	0	7.540820	-0.025242	-4.292237
45	6	0	5.574047	-0.025231	-3.411197
46	1	0	5.060231	-0.043524	-4.370215
47	6	0	4.861210	-0.011657	-2.220015
48	6	0	3.357458	-0.016812	-2.012733
49	6	0	3.237898	0.001869	-0.479810
50	6	0	-0.601958	-0.025022	-3.108270
51	6	0	-0.982802	-0.034151	-4.441329
52	6	0	-1.179393	-1.265559	-5.128350
53	1	0	-1.018944	-2.194524	-4.590982
54	6	0	-1.566394	-1.289118	-6.446812
55	1	0	-1.710882	-2.238155	-6.949310
56	6	0	-1.772163	-0.069582	-7.129687

57	6	0	-1.578286	1.166124	-6.463574
58	1	0	-1.744472	2.081573	-7.021597
59	6	0	-1.193277	1.185424	-5.150915
60	1	0	-1.044068	2.127013	-4.632997
61	6	0	-2.370021	-1.163087	-9.157023
62	1	0	-3.170068	-1.762250	-8.712544
63	1	0	-1.450934	-1.751663	-9.232096
64	1	0	-2.670746	-0.820109	-10.145714
65	6	0	-2.245002	-1.548111	9.013770
66	6	0	2.127236	1.152095	9.139275
67	6	0	3.477352	0.556384	8.707789
68	1	0	3.520418	-0.516442	8.928444
69	1	0	4.297386	1.048407	9.245164
70	1	0	3.655881	0.686897	7.635140
71	6	0	1.996821	0.982680	10.655823
72	1	0	1.070750	1.427028	11.038502
73	1	0	2.834503	1.485352	11.152695
74	1	0	2.020269	-0.072605	10.951895
75	6	0	2.112999	2.656741	8.823180
76	1	0	2.248486	2.848048	7.753361
77	1	0	2.923178	3.165069	9.360517
78	1	0	1.163547	3.110931	9.129505
79	6	0	-3.582231	-0.895579	8.626616
80	1	0	-3.611071	0.153574	8.943104
81	1	0	-4.414788	-1.420911	9.110639
82	1	0	-3.751285	-0.925474	7.544920
83	6	0	-2.121982	-1.510906	10.539650
84	1	0	-1.201966	-1.995513	10.886384
85	1	0	-2.967052	-2.046673	10.986948
86	1	0	-2.138053	-0.485073	10.925554

87	6	0	-2.252350	-3.020954	8.571793
88	1	0	-2.386475	-3.120992	7.489452
89	1	0	-3.072702	-3.559628	9.062091
90	1	0	-1.311585	-3.514530	8.841782
91	6	0	2.753367	-1.294055	-2.637590
92	1	0	1.668349	-1.277614	-2.490319
93	1	0	2.914943	-1.244118	-3.724920
94	6	0	3.300741	-2.615187	-2.106534
95	1	0	4.388413	-2.656849	-2.255295
96	1	0	3.133930	-2.670774	-1.022141
97	6	0	2.652390	-3.823712	-2.776323
98	1	0	2.813580	-3.769469	-3.863805
99	1	0	1.562657	-3.781267	-2.626798
100	6	0	3.177654	-5.158362	-2.256495
101	1	0	4.266812	-5.203792	-2.405880
102	1	0	3.017592	-5.212737	-1.169370
103	6	0	2.527797	-6.368287	-2.921207
104	1	0	2.687777	-6.313166	-4.007535
105	1	0	1.439807	-6.321343	-2.770879
106	6	0	3.058654	-7.696051	-2.393886
107	1	0	4.139374	-7.782686	-2.560247
108	1	0	2.575960	-8.547683	-2.886458
109	1	0	2.882400	-7.790326	-1.315450
110	6	0	2.743259	1.239617	-2.669317
111	1	0	2.900729	1.161019	-3.755611
112	1	0	1.659104	1.220542	-2.516783
113	6	0	3.282972	2.578805	-2.177154
114	1	0	3.116239	2.665018	-1.094738
115	1	0	4.370226	2.623443	-2.327958
116	6	0	2.626078	3.762474	-2.882105

117	1	0	1.536566	3.716112	-2.732272
118	1	0	2.788591	3.677602	-3.967404
119	6	0	3.140675	5.115839	-2.401564
120	1	0	2.978381	5.201539	-1.316764
121	1	0	4.229727	5.165040	-2.550686
122	6	0	2.482821	6.300226	-3.103339
123	1	0	1.394902	6.249038	-2.954013
124	1	0	2.645741	6.214135	-4.187184
125	6	0	3.001986	7.647284	-2.614751
126	1	0	2.822985	7.772011	-1.539861
127	1	0	2.513391	8.479979	-3.133186
128	1	0	4.082296	7.737684	-2.781886
